# Heart Mitochondrial Proteome Study Elucidates Changes in Cardiac Energy Metabolism and Antioxidant PRDX3 in Human Dilated Cardiomyopathy

**DOI:** 10.1371/journal.pone.0112971

**Published:** 2014-11-14

**Authors:** Esther Roselló-Lletí, Estefanía Tarazón, María G. Barderas, Ana Ortega, Manuel Otero, Maria Micaela Molina-Navarro, Francisca Lago, Jose Ramón González-Juanatey, Antonio Salvador, Manuel Portolés, Miguel Rivera

**Affiliations:** 1 Cardiocirculatory Unit, Health Research Institute Hospital La Fe, Valencia, Spain; 2 Department of Vascular Physiopathology, Hospital Nacional de Parapléjicos, SESCAM, Toledo, Spain; 3 Cellular and Molecular Cardiology Research Unit, Department of Cardiology and Institute of Biomedical Research, University Clinical Hospital, Santiago de Compostela, Spain; 4 Cardiology Service, Hospital La Fe, Valencia, Spain; 5 Cell Biology and Pathology Unit, Health Research Institute Hospital La Fe, Valencia, Spain; Scuola Superiore Sant'Anna, Italy

## Abstract

**Background:**

Dilated cardiomyopathy (DCM) is a public health problem with no available curative treatment, and mitochondrial dysfunction plays a critical role in its development. The present study is the first to analyze the mitochondrial proteome in cardiac tissue of patients with DCM to identify potential molecular targets for its therapeutic intervention.

**Methods and Results:**

16 left ventricular (LV) samples obtained from explanted human hearts with DCM (n = 8) and control donors (n = 8) were extracted to perform a proteomic approach to investigate the variations in mitochondrial protein expression. The proteome of the samples was analyzed by quantitative differential electrophoresis and Mass Spectrometry. These changes were validated by classical techniques and by novel and precise selected reaction monitoring analysis and RNA sequencing approach increasing the total heart samples up to 25. We found significant alterations in energy metabolism, especially in molecules involved in substrate utilization (ODPA, ETFD, DLDH), energy production (ATPA), other metabolic pathways (AL4A1) and protein synthesis (EFTU), obtaining considerable and specific relationships between the alterations detected in these processes. Importantly, we observed that the antioxidant PRDX3 overexpression is associated with impaired ventricular function. PRDX3 is significantly related to LV end systolic and diastolic diameter (r = 0.73, *p* value<0.01; r = 0.71, *p* value<0.01), fractional shortening, and ejection fraction (r = −0.61, *p* value<0.05; and r = −0.62, *p* value<0.05, respectively).

**Conclusion:**

This work could be a pivotal study to gain more knowledge on the cellular mechanisms related to the pathophysiology of this disease and may lead to the development of etiology-specific heart failure therapies. We suggest new molecular targets for therapeutic interventions, something that up to now has been lacking.

## Introduction

Heart failure (HF), a major and growing public health problem, is a current worldwide pandemic with an unacceptable high level of morbidity and mortality in industrialized countries and with no curative treatment currently available. Dilated cardiomyopathy (DCM), one of the most frequent causes of HF, is a severe pathology of unknown etiology characterized by impaired systolic function with increased ventricular mass, volume, and wall thickness [1], [2]. The mechanisms underlying the development of this cardiomyopathy are multiple, complex, and not well understood.

Mitochondria are the major energy production sites within cells [3]. Cardiac energy deficits have been reported in the failing heart, with convincing evidence of the important effect of mitochondrial dysfunction in the development and progression of HF in human and animal models resulting from its central role in energy production, metabolism, calcium homeostasis, oxidative stress, and cell death [4]–[8]. Some studies identify mitochondria as both the target and origin of major pathogenic pathways that cause myocardial dysfunction [9]. Nevertheless, the mitochondria-specific role and the proteins contributing to HF are unclear. In earlier studies, this organelle has been studied using experimental models and classic biochemical methods [10]–[12]. These studies usually focused on only one particular protein rather than the whole cardiac mitochondrial proteome, despite the fact that methods designed to enrich and purify the mitochondria represent one of the most long-standing examples of proteome subfractionation [13]–[15]. Thus, characterization of the mitochondrial proteome could provide new insight into cardiac dysfunction and suggest new molecular targets for the therapeutic intervention of DCM. However, the mitochondrial proteome has not been analyzed in pathological human hearts.

Here, we isolate mitochondria from left ventricular (LV) samples of explanted human hearts with DCM and use a proteomic approach to investigate the variations in mitochondrial protein expression. Our results identify the overexpression of several proteins involved mainly in energy metabolism but also in stress response and protein synthesis in dilated human hearts. We focus on seven representative mitochondrial proteins with different expressions in control (CNT) and diseased hearts validated by different classical techniques as well as novel and precise selected reaction monitoring (SRM) analysis and RNA sequencing (RNAseq) approach. We find that some proteins involved in the different components of cardiac energy metabolism and protein biosynthesis could have an important role in this cardiomyopathy. LV dysfunction is directly related with the antioxidant PRDX3 expression in DCM.

## Materials and Methods

### Ethics statement

The project was approved by the Ethics Committee of Hospital La Fe, Valencia, and all participants gave their written, informed consent. The study was conducted in accordance with the guidelines of the Declaration of Helsinki [16].

### Tissue sources

The experiments were performed using LV samples from explanted human hearts from Caucasian patients with DCM undergoing cardiac transplantation. Clinical history, hemodynamic study, electrocardiography, and Doppler echocardiography data were available from all of these patients. Non-ischemic DCM was diagnosed when patients had LV systolic dysfunction (ejection fraction, <40%) with a dilated non-hypertrophic LV (LV diastolic diameter, >55 mm) on echocardiography. Moreover, none of the patients had existing primary valvular disease or a familial history of DCM. All of the patients were functionally classified according to the New York Heart Association (NYHA) criteria and were receiving medical treatment following the guidelines of the European Society of Cardiology [17].

Non-diseased donor hearts were used as CNT samples. The hearts were initially considered for transplantation but were subsequently deemed unsuitable either because of blood type or size incompatibility. The cause of death was cerebrovascular or motor vehicle accident. All donors had normal LV function and no history of myocardial disease or active infection at the time of the transplantation.

Transmural samples were taken from the region around the apex of the left ventricle and stored at 4°C for a maximum of 6 h from the time of coronary circulation loss. The samples were stored at −80°C until the mitochondrial isolation was performed. Of the 25 heart samples, 16 were used in the proteomic analysis (DCM, n = 8; CNT, n = 8). The 25 heart samples were used in the validation to improve the numerical base with a higher number of patients (DCM, n = 17; CNT, n = 8).

### Mitochondrial isolation and proteomic analysis

Mitochondrial isolation was performed using standard homogenization, protease digestion, and differential centrifugation methods as previously described by Imahashi *et al*. [15]. The isolation was made from 50 mg of left ventricular tissue, obtaining a final protein concentration of 10 µg/µl.

### 2D–DIGE

Protein samples were precipitated using a 2-D Clean-Up Kit (GE Healthcare) as per the manufacturer’s protocol. Samples were labeled using CyDye DIGE Fluor Minimal Dyes (GE Healthcare) according to the manufacturer’s recommendations. The samples were resuspended in 20 µL of labeling buffer containing 7 M urea, 2 M thiourea, 4% CHAPS, and 30 mM Tris. The pH was then checked in every sample to ensure an optimal labeling reaction (pH 8.0–9.0). Aliquots (50 µg protein in 10 µL) were separated into individual tubes and a pooled internal standard was generated by mixing of equal amounts of all samples included in the experiment. A total of 400 pmol of the appropriate CyDye was added to each sample according to the experimental protocol. This experimental design included 2 experimental groups and 8 samples per group, and thus 16 samples were labeled with either Cy3 or Cy5. The labeling reactions were developed for 30 min on ice in the dark and then quenched by incubation with 1 µL of 10 mM lysine for 10 min in the dark. The labeled samples were combined according to the experimental design and loaded onto the same immobilized pH-gradient (IPG) strip. We randomized the CyDye assignments and sample combinations in each gel, which also included one aliquot of the pooled internal standard labeled with Cy2.

### Two-dimensional electrophoresis

The combined samples were diluted with a rehydration solution containing 8 M urea, 4% CHAPS, 20 mM DTT, and 1% IPG buffer (pH 3–11 NL; GE Healthcare) to a final volume of 450 µL. The IPG strips (24 cm; pH 3–11 NL) were allowed to rehydrate overnight in a re-swelling tray (GE Healthcare). After the strips were passively rehydrated, they were placed in an Ettan IPGphor Manifold ceramic tray (GE Healthcare) and isoelectric focusing (IEF) was performed in an Ettan IPGphor 3 unit (GE Healthcare) at 20°C according to the following program: 1-h gradient to 1000 V, 1.5-h gradient to 1500 V, 2-h gradient to 3500 V, 4-h constant at 3500 V, 2-h gradient to 6000 V, and, finally, constant at 6000 V to constitute a total of 60 kV/h. After IEF, the strips were equilibrated for 20 min in 1.5 M Tris (pH 8.8) buffer containing 6 M urea, 30% glycerol, 2% sodium dodecyl sulfate (SDS), and bromophenol blue to which 1% DTT had been added, followed by equilibration for 20 min more in the same buffer to which 2.5% iodoacetamide had been added. SDS–polyacrylamide gel electrophoresis (SDS-PAGE) was performed overnight as described by Laemmli [18] by using the Ettan DALT*six* Electrophoresis System (GE Healthcare) at 1 W/gel.

### Image acquisition and analysis

After SDS-PAGE, the gels were scanned with a Typhoon 9400 fluorescence gel scanner (GE Healthcare, Piscataway, NJ) using appropriate individual excitation and emission wavelengths, filters and photomultiplier (PTM) values that are sensitive for each of the Cy3, Cy5 and Cy2 dyes (PTM values: 480 nm, 490 nm, 500 nm, respectively). Relative protein quantification was performed on AS and healthy valves with DeCyder software v6.5 (GE Healthcare) and the multivariate statistical module EDA (Extended data analysis). The Differential in-gel analysis (DIA) module co-detected the 3 images of a gel (the internal standard and the two samples), measured the spot abundance in each image, and expressed these values as Cy3/Cy2 and Cy5/Cy2 ratios. These DIA datasets were then analysed using the Biological Variation Analysis module (BVA), which enabled the spot maps to be matched and the Cy3/Cy2 and Cy5/Cy2 ratios to be compared. Only protein spots with >1.5-fold differences in abundance were considered for the analysis. A statistical analysis was then carried out to determine the changes in protein species, with P-values below 0.05 accepted as significant when the Students t-test was applied. The gels were then re-stained with a silver staining kit (GE-Healthcare).

### In-gel protein digestion

Protein spots were excised manually and then automatically digested using the Ettan Digester (GE Healthcare). We used the digestion protocol previously described by Schevchenko *et al*. [19] with minor variations: the gel plugs were reduced using 10 mM DTT (Sigma-Aldrich; St. Louis, MO, USA) in 50 mM ammonium bicarbonate (99% purity; Scharlau) and alkylated using 55 mM iodoacetamide (Sigma-Aldrich) in 50 mM ammonium bicarbonate. The gel pieces were then rinsed with 50 mM ammonium bicarbonate in 50% methanol (gradient, high-performance liquid chromatography [HPLC] grade; Scharlau) and acetonitrile (gradient, HPLC grade; Scharlau) and dried in a Speedvac. Modified porcine trypsin (sequencing grade; Promega, Madison, WI, USA) at a final concentration of 20 ng/µL in 20 mM ammonium bicarbonate was added to the dry gel pieces, and the digestion was allowed to proceed at 37°C overnight. Finally, 60% aqueous acetonitrile and 0.5% trifluoroacetic acid (99.5% purity; Sigma-Aldrich) were added to extract peptides.

### MALDI-MS(/MS) and database searching

A total of 0.5 µL of each digestion solution was deposited using the thin-layer method onto a 384 Opti-TOF 123×81 mm MALDI plate (Applied Biosystems) and allowed to dry at RT. The same volume of matrix (3 mg/mL α-cyano-4-hydroxycinnamic acid (Sigma-Aldrich) in 60% acetonitrile/0.5% trifluoroacetic acid) was applied on every sample in the MALDI plate. MALDI-MS(/MS) data were obtained in an automated analysis loop by using a 4800 Plus MALDI TOF/TOF Analyzer (Applied Biosystems). Spectra were acquired in the reflector positive-ion mode by using an Nd:YAG 355-nm wavelength laser at 200 Hz laser frequency, and 1,000–2,000 individual spectra were averaged. The spectra were acquired uniformly by using fixed laser intensity. For the MS/MS 1-kV analysis mode, precursors were accelerated to 8 kV in Source 1 and selected using a relative resolution of 200 (FWHM) and metastable suppression. Fragment ions generated by collision with air in a CID chamber were further accelerated by 15 kV in Source 2. The mass data were automatically analyzed using the 4000 Series Explorer Software, Version 3.5.3 (Applied Biosystems). The MALDI-TOF mass spectra were internally calibrated using 2 trypsin autolysis ions with m/z = 842.510 and 2211.105. In the case of MALDI-MS/MS, calibrations were performed using the fragment-ion spectra obtained for Glub-fibrinopeptide (4700 Cal Mix; Applied Biosystems). MALDI-MS and MS/MS data were combined in the GPS Explorer Software Version 3.6 to search a non-redundant protein database (Swiss-Prot 2012_08) by using the Mascot software, version 2.2 (Matrix Science) [20], featuring 50 ppm precursor tolerance, 0.6-Da MS/MS fragment tolerance, carbamidomethyl cysteine as the fixed modification, and oxidized methionine as the variable modification, and allowing for 1 missed cleavage. The MALDI-MS (/MS) spectra and database search results were manually inspected in detail using the aforementioned software. In the case of the combined MS and MS/MS data, identifications were accepted when the confidence interval (CI%) calculated using the GPS software was ≥95%. Because Protein Scores and Ion Scores obtained from distinct searches cannot be directly compared, the GPS software calculates the CI% to combine the results of MS and MS/MS database searches. This coefficient value refers to a <5% probability of the observed match being a random event. In the case of the PMF spectra, identifications were accepted when the CI% was ≥99%.

### Gel electrophoresis and western blot analysis

Protein samples for the detection of pyruvate dehydrogenase E1 component subunit α, somatic form (ODPA), electron transfer flavoprotein-ubiquinone oxidoreductase (ETFD), dihydrolipoyl dehydrogenase (DLDH), delta-1-pyrroline-5-carboxylate dehydrogenase (AL4A1), ATP synthase subunit α (ATPA), elongation factor Tu (EFTU) and thioredoxin-dependent peroxide reductase (PRDX3) were separated using Bis-Tris electrophoresis on 4–12% polyacrylamide gels under reducing conditions. After the electrophoresis, the proteins were transferred from the gel to a polyvinylidene difluoride membrane using an iBlot Dry Blotting System (Invitrogen Ltd., UK) for western blot analyses. The primary detection antibodies used were anti-pyruvate dehydrogenase E1-alpha subunit mouse monoclonal antibody (1∶400), anti-EFTDH mouse monoclonal antibody (1∶400), anti-lipoamide dehydrogenase rabbit monoclonal antibody (1∶1000), anti-ATP5A mouse monoclonal antibody (1∶300), anti-TUFM rabbit polyclonal antibody (1∶2000), and anti-peroxiredoxin 3 mouse monoclonal antibody (1∶1000) (all obtained from Abcam, Cambridge, UK); and anti-ALDH4A1 mouse monoclonal antibody (1∶500) from Sigma-Aldrich. Anti-COX IV rabbit polyclonal antibody (1∶200) (Thermo Scientific, Rockford, IL, USA) was used as a loading control.

The bands were visualized using an acid phosphatase-conjugated secondary antibody and nitro blue tetrazolium/5-bromo-4-chloro-3-indolyl phosphate (NBT/BCIP, Sigma-Aldrich) substrate system. Finally, the bands were digitalized using an image analyzer (DNR Bio-Imagining Systems, Israel) and quantified using the GelQuant Pro (v12.2) program.

### Fluorescence microscopy

Human myocardial LV samples were fixed in 4% formalin, embedded in paraffin, cut into 5-µm sections, and mounted on superfrost glass slides. Sections were kept at 60°C overnight, deparaffinized with xylol followed by washing in 100%, 96%, 80%, and 70% ethanol. The samples were then blocked with phosphate buffered saline (PBS) containing 1% bovine serum albumin (BSA) for 15 min at RT. After blocking, the sections were incubated for 120 min at RT with the primary antibodies (described in the Western Blot Analysis section above) in the same buffer solution, and then with Alexa-conjugated secondary antibody (Invitrogen, USA) for 60 min at RT [21]. Finally, the sections were rinsed in PBS, mounted in Vectashield-conjugated 4′,6-diamidino-2-phenylindole (DAPI) for identifying the nucleous (Vector Laboratories, Burlingame, CA, USA), and examined under an Olympus BX50 fluorescence microscope (Tokyo, Japan). The images were processed by ImageJ (v. 1.4.3.67) software.

### Immunocytochemistry and electron microscopy

Myocardial samples (size 1 mm^3^) from the LV were fixed in a solution of 1.5% glutaraldehyde and 1% formaldehyde in 0.05 M cacodylate buffer (pH 7.4) for 1 h at 4°C. The samples were then post-fixed in 1% OsO_4_ for 1 h at 4°C, dehydrated in ethanol, and embedded in Epon 812. Ultra-thin sections measuring 80 nm were obtained and mounted on nickel grids and counter-stained with 2% uranyl acetate for 20 min and 2.7% lead citrate for 3 min [22], [23].

For immunogold labeling, ultra-thin sections were floated for 30 min on 0.1% BSA-Tris buffer (20 mM Tris-HCl, 0.9% NaCl [pH 7.4] containing 0.1% BSA, type V) and 2 h in a moist chamber at RT on sodium metaperiodate [24]. After being rinsed with bi-distilled water, the sections were incubated for 5 min with 3% H_2_O_2_. The grids were rinsed again with bi-distilled water and incubated separately in a moist chamber overnight at RT with the primary antibodies (described in the Western Blot Analysis section above) in the 0.1% BSA-Tris buffer. After being rinsed with 0.1% BSA-Tris buffer, the sections were incubated in a moist chamber for 1 h at 37°C with 0.1% BSA-Tris buffer (containing 0.05% Tween-20) and a goat anti-rabbit IgG-gold antibody (10 nm, 1∶10 dilution; Sigma) for DLDH and EFTU and a goat anti-mouse IgG-gold antibody (5 nm, 1∶10 dilution; Sigma) for ODPA, EFTD, AL4A1, ATPA, and PRDX3.

After rinses with 0.1% BSA-Tris buffer and bi-distilled water, the sections were air dried and counterstained first with uranyl acetate for 30 min and then with lead citrate for 5 s. Finally, the grids were air dried completely. For the electron microscopy observation, a Philips CM-100 was used, with magnifications ranging X4500–15000.

### Selected reaction monitoring (SRM)

Protein samples were reduced by incubating them with 100 mM DTT (Sigma Aldrich) in 50 mM ammonium bicarbonate (99% purity; Scharlau) for 30 min at 37°C. After reduction, alkylation with 55 mM iodoacetamide (Sigma Aldrich) in 50 mM ammonium bicarbonate was conducted for 20 min at RT. Next, we added 50 mM ammonium bicarbonate, 15% acetonitrile (LCMS grade, Scharlau), and, finally, sequencing-grade modified porcine trypsin (Promega) at a final ratio of 1 µg trypsin: 50 µg protein. After digestion at 37°C overnight, 2% formic acid (99.5% purity; Sigma Aldrich) was added and samples were cleaned using Pep-Clean spin columns (Pierce) according to the manufacturer’s instructions. Tryptic digests were dried in a Speedvac and resuspended in 2% acetonitrile/2% formic acid prior to MS analysis.

The LC-MS/MS system consisted of a TEMPO nano LC system (Applied Biosystems) combined with a nano LC Autosampler and coupled to a modified triple quadrupole (Applied Biosystems 4000 QTRAP LC/MS/MS System). Three replicate injections (4 µL containing 8 µg of protein) were made for each sample (except 2 samples with only 1 injection per sample) by using mobile phase A (2% ACN/98% water, 0.1% FA) at a flow rate of 10 µL/min for 5 min. Peptides were loaded onto a µ-Precolumn Cartridge (Acclaim Pep Map 100 C18; 5 µm, 100Å; 300 µm i.d. ×5 mm, LC Packings) to preconcentrate and desalt samples. Reversed-phase LC was performed on a C18 column (Onyx Monolithic C18; 150×0.1 mm i.d., Phenomenex) in a gradient of phase A and phase B (98% ACN/2% water, 0.1% FA). Peptides were eluted at a flow rate of 900 nL/min by following these steps: 2–15% B for 2 min, 15–30% B for 18 min, 30–50% B for 5 min, 50–90% B for 2 min, and, finally, 90% B for 3 min. The column was then regenerated with 2% B for another 15 min. Both TEMPO nano LC and 4000 QTRAP system were controlled using the Analyst Software, v.1.4.5. Theoretical SRM transitions were designed using MRMpilot software v1.1 (ABSciex), with the following settings: Enzyme = trypsin, missed cleavages = 0; modifications in peptide ≤3; charge states = +1 from 300 to 600 Da, +2 from 500 to 2000 Da, +3 from 900 to 3000 Da, +4 from 1600 to 4000 Da, +5 from 2400 to 10,000 Da; studied modification = none; fixed modifications = carboxyamidomethylation; variable modifications = none; min. number of amino acids ≥5; max. number of amino acids ≤30; ignore multiple modification sites; 3 transitions per peptide (Table_S2 in File S1). A pool containing a mixture of all the samples was digested as described previously and analyzed in the 4000QTrap using a MIDAS acquisition method that included the theoretical transitions. Transitions were selected when the three co-eluting peaks (corresponding to the three transitions of the same peptide) had a signal-to-noise ratio over 5 and the MS/MS data matched the theoretical spectrum for that peptide.

The mass spectrometer was set to operate in the positive-ion mode with an ion-spray voltage of 2800 V and a nanoflow interface heater temperature of 150°C. Source gas 1 and curtain gas were set to 20 and 20 psi, respectively, and nitrogen was applied as both curtain and collision gases. Collision energy was optimized to obtain maximal transmission efficiency and sensitivity for each SRM transition. A total of 42 MRM transitions (3 per peptide) were monitored during the analysis of each sample and were acquired at unit resolution in both Q1 and Q3, with dwell times of 20 and 50 ms that resulted in a cycle time of 1.2303 s. The IntelliQuan algorithm included in the Analyst 1.4.5 software was used to calculate abundances based on the peak areas after integration.

To carry out this analysis we pooled the samples (four samples per pool). Correlations between proteins were evaluated by calculating Pearson product–moment correlation coefficient of every transition from every peptide analyzed for each protein with respect to every transition of the peptides of the other proteins. If the results of 1 out of the 3 assayed transitions were divergent from the other two, this transition was not considered. While evaluating correlations, when 2 or more peptides were measured, the most significant peptide was selected, while the second one was used as a qualifier for the correlation, which was rejected if this second peptide had divergent results. Pearson's correlation coefficient (r) was calculated as a mean of all coefficients from all transitions from the most significant peptide-to-peptide correlation. When any of the transition-to-transition correlations was not significant, but close to signification, the greater transition-to-transition p value of the most significant peptide-to-peptide correlation was considered.

### RNA extraction

Heart samples were homogenized in TRIzol reagent in a TissueLysser LT (Qiagen, UK). All RNA extractions were performed using a PureLink Kit according to the manufacturer’s instructions (Ambion Life Technologies, CA, USA). RNA was quantified using a NanoDrop1000 spectrophotometer (Thermo Fisher Scientific, UK), and the purity and integrity of the RNA samples were measured using an Agilent 2100 Bioanalyzer with an RNA 6000 Nano LabChip kit (Agilent Technologies, Spain). All samples showed a 260/280 ratio of >2.0 and an RNA integrity number of ≥9.

### RNAseq

The RNA samples were isolated using a MicroPoly(A) Purist Kit (Ambion, USA). The total polyA-RNA samples were used to generate whole transcriptome libraries that were sequenced on a SOLiD 5500XL platform as per the manufacturer’s recommendations (Life Technologies, CA). The amplified cDNA quality was analyzed using the Bioanalyzer 2100 DNA 1000 kit (Agilent Technologies, Spain), and the cDNA was quantified using the Qubit 2.0 Fluorometer (Invitrogen, UK). Whole transcriptome libraries were used to generate SOLiD templated beads by following the SOLiD Templated Bead Preparation guide. Bead quality was estimated based on WFA (workflow analysis) parameters. The samples were sequenced using the 50625 paired-end protocol, which generated 75 nt+35 nt (Paired-End) +5 nt (Barcode) sequences. Quality data were measured using the SETS software parameters (SOLiD Experimental Tracking System).

### Computational analysis of RNAseq data

The initial whole transcriptome paired-end reads obtained from the sequencing were mapped against the latest version of the human genome (Version GRchr37/hg19) by using the Life Technologies mapping algorithm (http://www.lifetechnologies.com/). The aligned records were reported in the BAM/SAM format [25]. Bad quality reads (Phred score <10) were eliminated using the Picard Tools software [26].

The isoform and gene predictions were subsequently estimated using the cufflinks method [27], and the expression levels were calculated using the HTSeq software [28]. The Edge method was applied to analyze the differential expression between conditions [29]. This method relies on a Poisson model to estimate the RNAseq data variance for differential expression. We selected genes and isoforms that were calculated to exhibit *p* value<0.05 and fold-change >1.5.

### Statistics

Data are presented as mean ± standard deviation (SD). The Kolmogorov–Smirnov test was used to analyze the normal distribution of the variables. Comparisons between 2 groups were performed using Student’s *t*-test, while Pearson’s correlation coefficient was calculated to analyze the association between variables. Analyses were considered significant when *p* value<0.05. All statistical analyses were performed using SPSS software v. 20 for Windows (IBM SPSS Inc., Chicago, IL, USA).

## Results

### Patients’ clinical characteristics

LV tissue samples were obtained from 17 patients with DCM (82% men; mean age, 54±11 years; ejection fraction, <40%). These patients had a New York Heart Association (NYHA) functional classification of III–IV and were previously diagnosed with significant comorbidities including hypertension, and diabetes mellitus. The patients’ clinical and echocardiographic characteristics are summarized in Table 1. Eight non-diseased donor hearts were used as CNT samples (63% men; mean age, 55±8 years; ejection fraction, >50%).

**Table 1:** Clinical and echocardiographic characteristics of heart failure patients.

	DCM (n = 17)
Age (years)	54±11
Gender male (%)	82
BMI (kg/m^2^)	26±5
Prior hypertension (%)	29
Diabetes mellitus (%)	29
NYHA class	3.3±0.4
Hemoglobin (mg/dL)	13±2
Hematocrit (%)	40±7
Total cholesterol (mg/dL)	144±43
Duration of disease (months)	85±69
Echo-Doppler study	
Ejection fraction (%)	24±7
Fractional shortening (%)	13±4
Left ventricular end systolic diameter (mm)	66±13
Left ventricular end diastolic diameter (mm)	75±13
Left ventricle mass (g)	484±132
Left ventricle mass index (g/cm^2^)	255±80

Duration of disease from diagnosis of heart failure until heart transplant. BMI, body mass index; DCM, dilated cardiomyopathy; NYHA, New York Heart Association.

### Differentially expressed mitochondrial proteins in patients with HF of dilated etiology

The protein expressions of purified heart mitochondria from 8 DCM patients and 8 CNT were compared using two-dimensional differential gel electrophoresis (2D-DIGE). Each gel contained the mitochondrial proteome of DCM patients, CNT samples, and an internal standard. Gel images were imported into DeCyder Differential Analysis Software, which detected 1,171–1,418 protein spots. Reproducibility was tested by comparing the variation within the different gels in the same group. The *t*-test statistical analysis did not show significant differences. We focused on the identification of up- and downregulation of spot intensities where the fold change was ≥1.5 (*p* value<0.05). Considering these criteria, statistical analysis of the data in the DeCyder software revealed changes in the abundance of 19 protein spots corresponding to 17 mitochondrial proteins revealed by mass spectrometry. We encountered 16 significantly upregulated and 3 downregulated spots in DCM hearts (Figure_S1 in File S1). The tandem mass spectrometry (MS/MS) identification details are summarized in Table_S1 in File S1.

Table 2 shows that most of the proteins altered in the mitochondrial proteome of DCM patients are involved in cardiac energy metabolism, some implicated in substrate utilization such as ETFD or DLDH, while others are implicated in energy production such as ATPA. The remaining identified mitochondrial proteins were basically structural or implicated in the protein synthesis and stress response, such as coiled-coil-helix-coiled-coil-helix domain-containing protein 3, EFTU, and PRDX3, respectively. The majority of the altered proteins belong to the mitochondrial matrix or the inner membrane.

**Table 2:** Mitochondrial proteins differentially regulated in dilated cardiomyopathy *vs.* controls.

Spot	Accesion code	Protein name	Fold-change	*p*-value	MainLocalization	Function
324	ETFD_HUMAN	Electron transferflavoprotein-ubiquinoneoxidoreductase	+1.74	0.047	Inner membrane	Metabolism/Transport
335	CH60_HUMAN	60 kDa heat shockprotein	+1.95	0.023	Matrix	Stress response
344	ETFD_HUMAN	Electron transferflavoprotein-ubiquinoneoxidoreductase	+1.77	0.001	Matrix/Inner membrane	Metabolism/Transport
348	DLDH_HUMAN	Dihydrolipoyldehydrogenase	+2.25	0.008	Matrix	Metabolism
361	AL4A1_HUMAN	Delta-1-pyrroline-5-carboxylatedehydrogenase	+1.79	0.002	Matrix	Metabolism
407	DLDH_HUMAN	Dihydrolipoyldehydrogenase	+1.73	0.019	Matrix	Metabolism
472	ODO2_HUMAN	Dihydrolipoyllysine-residue succinyltransferasecomponent of 2-oxoglutaratedehydrogenasecomplex	+1.51	0.0001	Matrix	Metabolism
601	ODPA_HUMAN	Pyruvatedehydrogenase E1component subunitalpha, somatic form	+2.21	0.001	Matrix	Metabolism
604	ACADM_HUMAN	Medium-chainspecific acyl-CoAdehydrogenase	+1.79	0.027	Matrix	Metabolism
	ODPA_HUMAN	Pyruvatedehydrogenase E1component subunitalpha, somatic form			Matrix	Metabolism
614	KCRS_HUMAN	Creatine kinaseS-type	−2.01	0.001	Innermembrane	Metabolism
689	EFTU_HUMAN	Elongation factorTu	+2.41	0.008	Mitochondrion	Protein biosynthesis
732	MDHM_HUMAN	Malatedehydrogenase	−1.75	0.008	Matrix	Metabolism
861	CY1_HUMAN	Cytochrome c1,heme protein	+1.71	0.007	Innermembrane	Metabolism/Respiratory chain
	ECH1_HUMAN	Delta(3,5)-Delta(2,4)-dienoyl-CoA isomerase			Matrix	Metabolism
882	ATPA_HUMAN	ATP synthasesubunit alpha	+2.23	0.01	Innermembrane	Metabolism/Respiratory chain
904	ATPA_HUMAN	ATP synthasesubunit alpha	+1.93	0.049	Inner membrane	Metabolism/Respiratory chain
960	CHCH3_HUMAN	Coiled-coil-helix-coiled-coil-helixdomain-containingprotein 3	+1.79	0.006	Innermembrane	Structural
1019	PRDX3_HUMAN	Thioredoxin-dependent peroxidereductase	+1.73	0.031	Mitochondrion	Stress response
	NDUV2_HUMAN	NADHdehydrogenase [ubiquinone]flavoprotein 2			Innermembrane	Metabolism/Respiratory chain
1039	PRDX3_HUMAN	Thioredoxin-dependent peroxidereductase	+1.59	0.046	Mitochondrion	Stress response
1140	PRDX5_HUMAN	Peroxiredoxin-5	−1.89	0.023	Mitochondrion	Stress response

### Validation of protein differential abundance and mRNA levels

**Figure 1:**
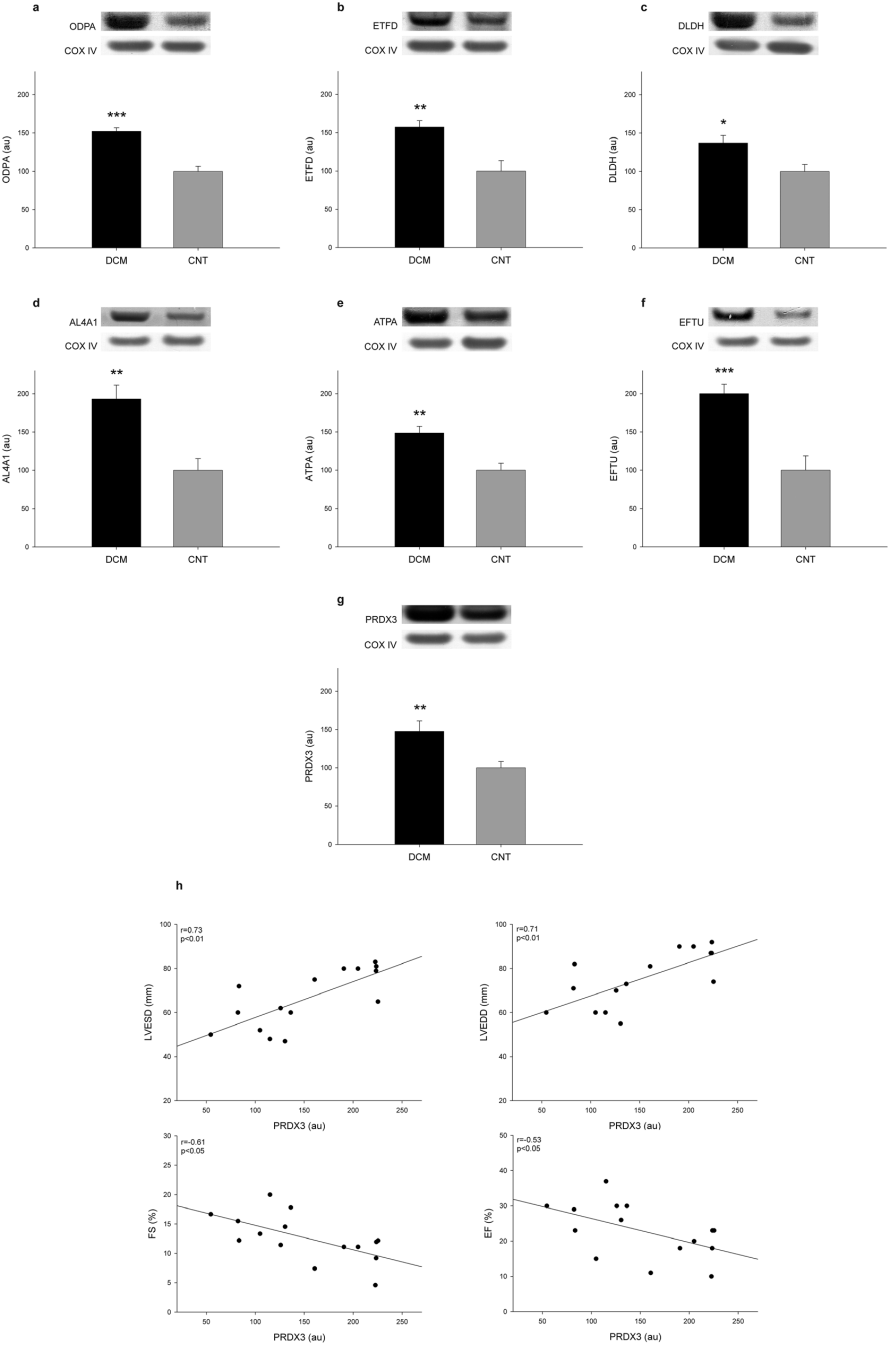
Mitochondrial protein overexpression in dilated human hearts and relationship between PRDX3 and left ventricular function. (a–g) The influence of dilated cardiomyopathy on the amount of each representative protein involved in cardiac energy metabolism (ODPA, ETFD, DLDH, AL4A1, and ATPA), protein biosynthesis (EFTU), and stress response (PRDX3) analyzed using western blotting techniques. As shown, all proteins were significantly increased in the DCM group (n = 17) compared with the CNT group (n = 8). The values from the controls were set to 100. Values were normalized to COX IV and finally to the CNT group. The data are expressed as mean+SEM in arbitrary units (optical density). Images are representative of the results obtained for all of the patients with DCM and the CNT included in the study. (h) Scatter plots showing the relationship between PRDX3 protein levels and left ventricular function, specifically with fractional shortening, left ventricular end systolic diameter, and left ventricular end diastolic diameter. CNT, control; DCM, dilated cardiomyopathy; ODPA, pyruvate dehydrogenase E1 component subunit α, somatic form; ETFD, electron transfer flavoprotein-ubiquinone oxidoreductase; DLDH, dihydrolipoyl dehydrogenase; AL4A1, delta-1-pyrroline-5-carboxylate dehydrogenase; ATPA, ATP synthase subunit α; EFTU, elongation factor Tu; PRDX3, thioredoxin-dependent peroxide reductase; FA, fractional shortening; LVESD, left ventricular end systolic diameter; LVEDD, left ventricular end diastolic diameter. *p value<0.05, **p value<0.01, ***p value<0.0001.

A selection of representative proteins of each mitochondrial function and/or localization and involved in heart damage was validated using different techniques that compared its levels among DCM patients (n = 17) and CNT (n = 8). Therefore, using western blot techniques, we determined the levels of some relevant proteins involved in metabolism, including ODPA, ETFD, DLDH, AL4A1, ATPA; protein synthesis EFTU; and stress response PRDX3. As shown in Figures 1a-g, levels of all of the analyzed molecules were significantly increased in the pathological samples (ODPA, 152±19 vs. 100±18 au, *p* value<0.0001; ETFD, 157±35 vs. 100±37 au, *p* value<0.01; DLDH, 132±42 vs. 100±25 au, *p* value<0.05; AL4A1, 193±75 vs. 100±43 au, *p* value<0.01; ATPA, 149±36 vs. 100±25 au, *p* value<0.01; EFTU, 200±51 vs. 100±53 au, *p* value<0.0001; and PRDX3, 148±57 vs. 100±23 au, *p* value<0.01). These results coincided with those of the proteomic analysis.

We determined whether there was a relationship between these protein levels and the clinical characteristics shown in Table 1. The LV function parameters were completely available in 15 of 17 samples from DCM patients. We found significant relationships between PRDX3 levels and LV function (Fig. 1h). The results obtained showed that PRDX3 is significantly correlated with LV end systolic diameter, LV end diastolic diameter, fractional shortening, and ejection fraction (r = 0.73, *p* value<0.01; r = 0.71, *p* value<0.01; r = −0.61, *p* value<0.05; and r = −0.62, *p* value<0.05, respectively).

**Figure 2:**
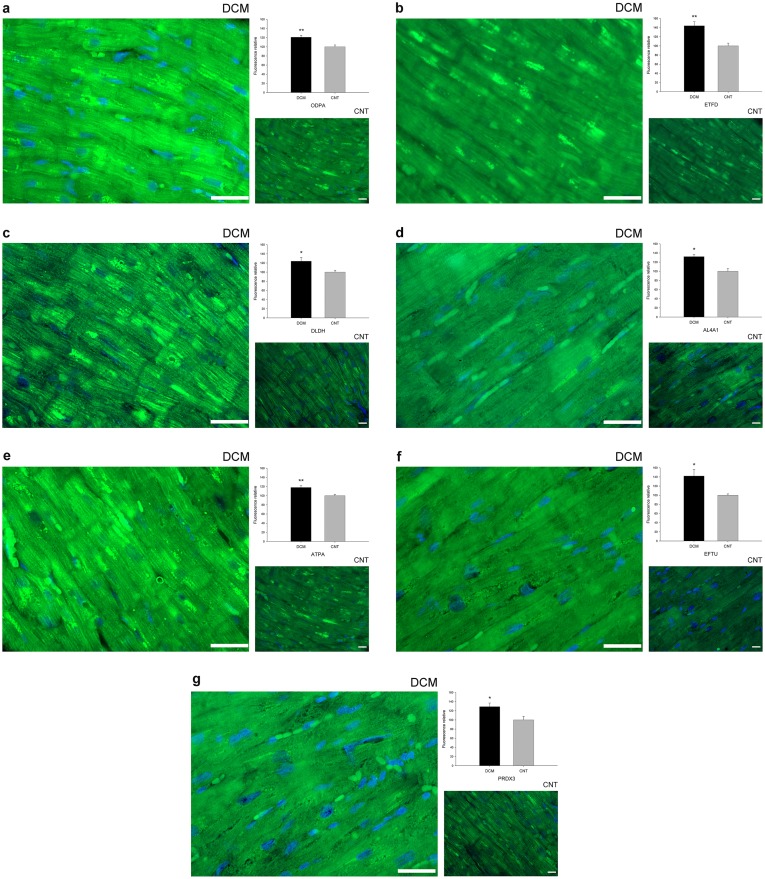
Mitochondrial protein overexpression in dilated human hearts according to immunofluorescence techniques. Influence of dilated cardiomyopathy on the amount of each representative protein involved in cardiac energy metabolism (ODPA, ETFD, DLDH, AL4A1, and ATPA), protein biosynthesis (EFTU), and the stress response (PRDX3). Immunofluorescence of (a) ODPA, (b) ETFD, (c) DLDH, (d) AL4A1, (e) ATPA, (f) EFTU, and (g) PRDX3 were significantly increased in patients with dilated cardiomyopathy compared with the control group. Here we show the nucleus co-stained with DAPI (blue). All of the micrographs are representative of the results obtained in four independent experiments for each group and protein studied, DCM (n = 4) and CNT (n = 4). The bar represents 100 µm. The bar graph shows the relative fluorescence intensity in dilated compared to control hearts. The data are expressed as mean ± SEM. CNT, control; DCM, dilated cardiomyopathy; ODPA, pyruvate dehydrogenase E1 component subunit α, somatic form; ETFD, electron transfer flavoprotein-ubiquinone oxidoreductase; DLDH, dihydrolipoyl dehydrogenase; AL4A1, delta-1-pyrroline-5-carboxylate dehydrogenase; ATPA, ATP synthase subunit α; EFTU, elongation factor Tu; PRDX3, thioredoxin-dependent peroxide reductase. *p value<0.05, **p value<0.01.

The immunofluorescence study findings were consistent with the increased levels observed by western blotting and proteomic analysis showing that the intensity of all validated proteins was higher in the dilated hearts than in the CNT samples. These proteins had a diffuse cytoplasmic distribution with a significantly higher percentage of fluorescence in the dilated group (ODPA, 21%, *p* value<0.01 [Fig. 2a]; ETFD, 44%, *p* value<0.01 [Fig. 2b]; DLDH, 24%, *p* value<0.05 [Fig. 2c]; AL4A1, 32%, *p* value<0.01 [Fig. 2d]; ATPA, 18%, *p* value<0.01 [Fig. 2e]; EFTU, 42%, *p* value<0.05 [Fig. 2f]; PRDX3, 29%, *p* value<0.05 [Fig. 2g]).

**Figure 3:**
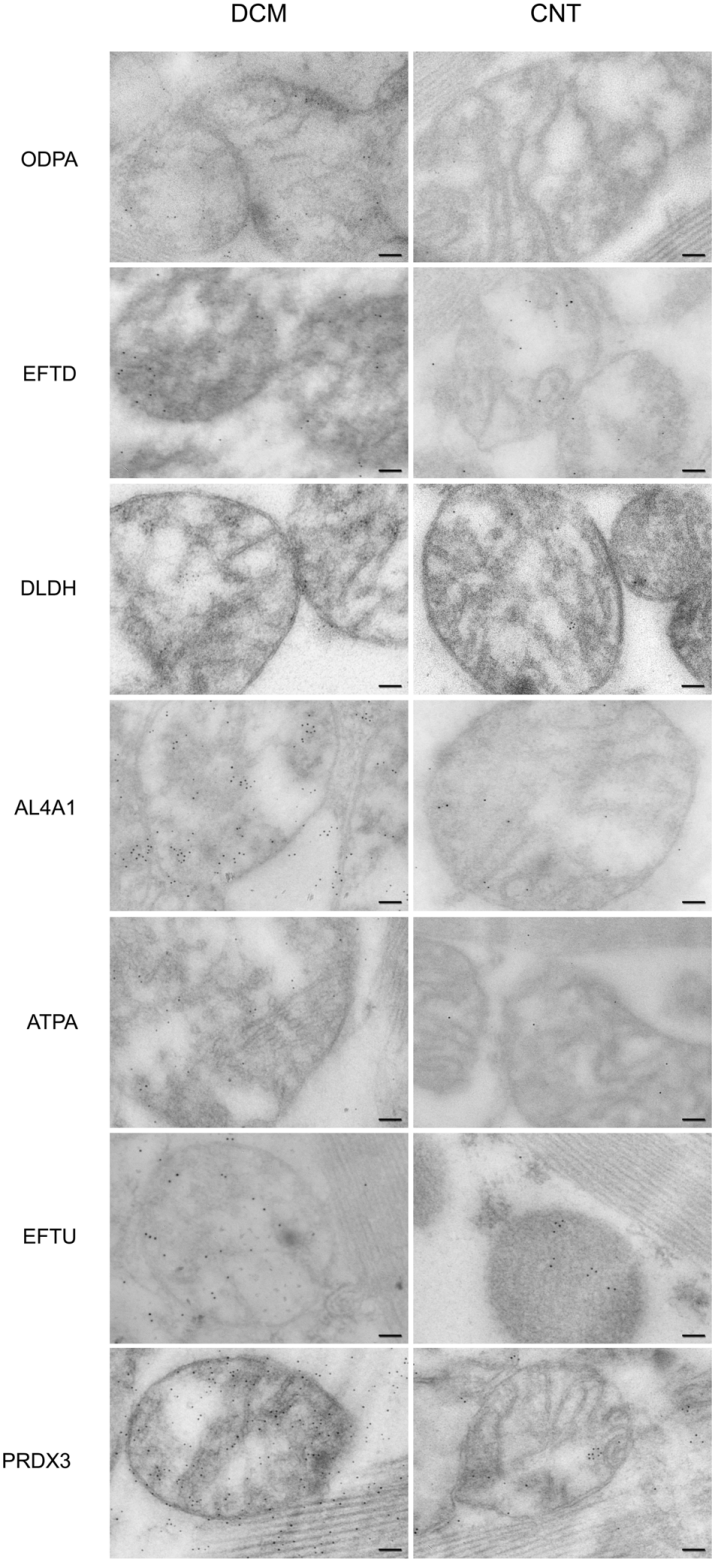
Mitochondrial protein localization and overexpression in dilated human hearts analyzed using transmission electron microscopy. Influence of dilated cardiomyopathy on the amount and localization of the representative proteins involved in cardiac energy metabolism (ODPA, ETFD, DLDH, AL4A1, and ATPA), protein biosynthesis (EFTU), and stress response (PRDX3). We observed an increase in immunogold labeling in all proteins studied in DCM hearts. We also confirmed the location of all proteins analyzed and observed a similar distribution of each protein upon comparing pathological with control samples. The bar represents 100 nm. CNT, control; DCM, dilated cardiomyopathy; ODPA, pyruvate dehydrogenase E1 component subunit α, somatic form; ETFD, electron transfer flavoprotein-ubiquinone oxidoreductase; DLDH, dihydrolipoyl dehydrogenase; AL4A1, delta-1-pyrroline-5-carboxylate dehydrogenase; ATPA, ATP synthase subunit α; EFTU, elongation factor Tu; PRDX3, thioredoxin-dependent peroxide reductase.

Immunocytochemistry studies confirmed the previous results and the localization or distribution of these mitochondrial proteins. Figure 3 shows an increase in immunogold labeling in ODPA, ETFD, DLDH AL4A1, ATPA, EFTU, and PRDX3 in DCM hearts compared to CNT hearts. We observed similar localization of each protein in the pathological and CNT samples.

To validate the previous analyses and to evaluate the possible relationship between the altered proteins involved in the different mitochondrial processes, these molecules were monitorized by SRM (Table_S2 and S3 in File S1). Differential expression was confirmed in all three transitions per peptide (*p* value<0.05). We obtained a good relationship between the abundance of altered proteins involved in substrate utilization (ODPA vs. DLDH, r = 0.58, *p* value<0.05; ODPA vs. ETFD, r = 0.65, *p* value<0.01; DLDH vs. ETFD, r = 0.59, *p* value<0.05) and with other altered molecules implicated in the metabolic process (ODPA vs. AL4A1, r = 0.73, *p* value<0.001; DLDH vs. AL4A1, r = 0.58, *p* value<0.05; ETFD vs. AL4A1, r = 0.76, *p* value<0.01) and protein synthesis (ODPA vs. EFTU, r = 0.66, *p* value<0.01; DLDH vs. EFTU, r = 0.74, *p* value<0.001; ETFD vs. EFTU, r = 0.67, *p* value<0.01). In addition, these molecules showed a relevant correlation with ATPA (ODPA vs. ATPA, r = 0.77, *p* value<0.001; DLDH vs. ATPA, r = 0.74, *p* value<0.001; ETFD vs. ATPA, r = 0.61, *p* value<0.01; AL4A1 vs. ATPA, r = 0.78, *p* value<0.001; EFTU vs. ATPA, r = 0.89, *p* value<0.0001).

**Figure 4:**
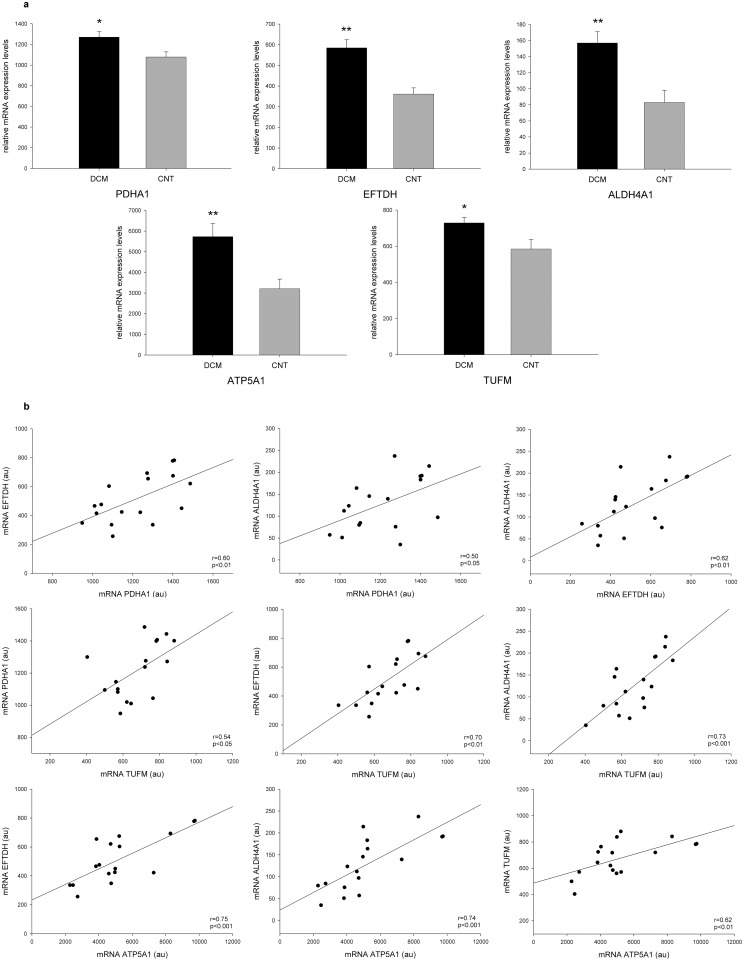
Levels of mRNA expression determined by RNAseq. (a) mRNA levels of the ODPA gene (*PDHA1*), ETFD gene (*ETFDH*), AL4A1 gene (*ALDH4A1*), ATPA gene (*ATP5A1*), and EFTU gene (*TUFM*) were increased in the dilated group compared to the control group. The data are expressed as mean ± SEM in mRNA relative expression. (b) The scatter plots show the relationship between the mRNA expression of molecules altered involved in substrate utilization (*PDHA1* and *ETFDH*) and also with other altered protein implicated in metabolic processes (*ALDH4A1*) and protein synthesis (*TUFM*). These genes showed a relevant correlation with *ATP5A1*, which is involved in energy production. CNT, control; DCM, dilated cardiomyopathy; ODPA, pyruvate dehydrogenase E1 component subunit α, somatic form; ETFD, electron transfer flavoprotein-ubiquinone oxidoreductase; AL4A1, delta-1-pyrroline-5-carboxylate dehydrogenase; ATPA, ATP synthase subunit α; EFTU, elongation factor Tu. *p value<0.05, **p value<0.01.

The mRNA differences between DCM patients and CNT were determined by RNAseq. The mRNA levels of the ODPA (*PDHA1*), ETFD (*ETFDH*), AL4A1 (*ALDH4A1*), ATPA (*ATP5A1*), and EFTU (*TUFM*) genes were increased in the DCM group (18-fold, *p* value<0.05; 62-fold, *p* value<0.01; 88-fold, *p* value<0.01; 79-fold, *p* value<0.01; and 25-fold, *p* value<0.05, respectively) (Fig. 4a). These results also coincided with those of proteomic analysis demonstrating the same tendency between gene expression and protein levels. However, the DLDH (*DLD*) and PRDX3 (*PRDX3*) gene expressions did not reach statistical significance. Finally, we obtained a good relationship between the mRNA expression of altered molecules involved in substrate utilization (*PDHA1* vs. *ETFDH*, r = 0.60, *p* value<0.01) and with other altered genes implicated in the metabolic process (*PDHA1* vs. *ALDH4A1*, r = 0.50, *p* value<0.05; *ETFDH* vs. *ALDH4A1*, r = 0.62, *p* value<0.01) and protein synthesis (*PDHA1* vs. *TUFM*, r = 0.54, *p* value<0.05; *ETFDH* vs. *TUFM,* r = 0.70, *p* value<0.01). In addition, these molecules showed a relevant correlation with *ATP5A1* (*ETFDH* vs. *ATP5A1*, r = 0.75, *p* value<0.001; *ALDH4A1* vs. *ATP5A1*, r = 0.62, *p* value<0.001; *TUFM* vs. *ATP5A1*, r = 0.70, *p* value<0.001) (Fig. 4b).

## Discussion

### Mitochondrial proteome of DCM human hearts

In the present study, we carried out a 2D-DIGE analysis of isolated cardiac mitochondria from the LV tissue of DCM patients to investigate mitochondrial cardiac protein expression changes. The mechanisms responsible for mitochondrial dysfunction in human hearts are poorly understood and the animal models used to elucidate it may not reflect the true pathophysiology of DCM. There are some studies based on proteomic approach in failing hearts [30]–[33], however this is the first study to analyze the mitochondrial proteome in pathological human hearts. As described so far, isolating and analyzing single organelles is a right approach to uncover essential information regarding their regulation and interplay [13].

Because the heart consumes more ATP than any other organ, it is particularly rich in mitochondria. During HF development, both energy demands and metabolism change show a remarkable decrease in oxidative phosphorylation and a shift toward glucose over fatty acid utilization [5]. Mitochondria are a major source and target of free radicals, and the collapse of the mitochondrial transmembrane potential can initiate the signaling cascades involved in apoptosis [34]. They also play an important role in antioxidant regeneration and are responsible for the majority of ATP production [35]. A recent study by Ahuja *et al*. showed that DCM is clearly associated with enhanced mitochondrial biogenesis and mtDNA deletion, making evident the essential role of mitochondria in this form of HF [36]. Thus, analysis of the mitochondrial proteome could provide new insights into cardiac dysfunction in DCM patients. Here, we identified 19 protein spots corresponding to 17 mitochondrial proteins altered in failing hearts (14 increased, 3 decreased). These changes comprise many aspects of mitochondrial function, including metabolism, transport, respiratory chain, stress response, protein synthesis, and cell death.

### Alterations in substrate utilization in DCM human hearts

Impaired cardiac energy metabolism is known to play a major role in HF [4], [6]. Our results are in concordance with these studies, as we found that most of the proteins altered in the mitochondrial proteome of DCM patients are involved in cardiac energy metabolism, 12 of the 17 differentially regulated proteins identified (70%) are involved in this process. The metabolic machinery has three components: substrate utilization, energy production, and energy transfer and utilization. The main way that myocytes compensate for decreasing mitochondrial function is by increasing glycolysis-related protein expression [5]. Therefore, we focused on three overexpressed proteins involved in substrate utilization. The pyruvate dehydrogenase complex is a multi-enzyme system composed of multiple copies of 3 catalytic components, E_1_ (E_1_α and E_1_β), E_2_, and E_3_. Specifically, ODPA catalyzes the overall conversion of pyruvate to acetyl-CoA and CO_2_, and thereby links the glycolytic pathway to the tricarboxylic cycle [37], [38]. We found that levels of this protein and its mRNA are significantly increased in DCM in concordance with the earlier study results of a canine HF model [39]. In addition, we found that ETFD was overexpressed. Electron transfer flavoproteins are heterodimeric proteins that transfer electrons between primary dehydrogenases and respiratory chains and link the oxidation of fatty acids and some amino acids to the mitochondrial respiratory system [40], [41]. Finally, we also validated the overexpression of DLDH, a stable homodimer and essential component of the pyruvate dehydrogenase and glycine cleavage system as well as the α-ketoacid dehydrogenase complex [42]. This result is consistent with those published by our group and also by Li *et al.* in previous studies of total homogenate of LV tissue of DCM patients observing increased DLDH levels [43], [44]. In addition, we found a good correlation between the protein levels and mRNA expression of these molecules and also with other altered proteins and its mRNA levels implicated in metabolic process and protein synthesis, specifically AL4A1 and EFTU, thereby interconnecting the alterations found in different processes.

### Alterations in energy production in DCM human hearts

High myocardial energy production rates are required to maintain the constant demand of the working heart ATP and alterations in oxidative phosphorylation reduce cardiac function by providing an insufficient supply of ATP to cardiomyocytes [5]. Although the activity of electron transport chain complexes and ATP synthase (the complex responsible for ATP production) activity are known to be reduced in HF [45], reports on the individual levels of ATP synthase subunits in this syndrome are contradictory. While some authors observed that ATP synthase levels did not change [45] or diminish [46] in failing hearts, other studies revealed an increase in ATPA [47], [48]. In the present work, we observed a significant overexpression of this protein. This lack of agreement between studies might be because of differences in the sample type or protocols and techniques used to detect these proteins. We also found a significant positive correlation of ATPA protein levels and mRNA expression with the overexpressed molecules involved in substrate utilization, highlighting the relationship between two principal components of the cardiac energy metabolism system. In other words, changes in some proteins involved in substrate utilization implicate modifications in specific components of oxidative phosphorylation. In addition, we found a good correlation between ATPA protein levels and mRNA expression with EFTU and *TUFM*, respectively. Thus, alterations in the energy production system are linked to higher activation of protein biosynthesis and oxidative damage in failing hearts since EFTU promotes the GTP-dependent binding of aminoacyl-tRNA to the A-site of ribosomes, and its downregulation increase reactive oxygen species [49].

### Antioxidant PRDX3 overexpression is associated with impaired ventricular function

A large number of studies have reported that oxidative stress is important in the pathophysiology and development of HF via free radical production [50], [51]. Reactive oxygen species play a key role in the onset and progression of coronary heart disease, tissue necrosis, and contractile dysfunction [52], [53]. PRDX3 is a mitochondrial antioxidant protein that protects radical-sensitive enzymes against oxidative damage by a radical-generating system. Matsusshima *et al.* reported that PRDX3 overexpression protects the heart against post-myocardial infarct remodeling and failure in mice, reducing LV cavity dilation, dysfunction, fibrosis, and apoptosis [54]. These results are consistent with our findings, since we found a significant increase in the protein levels of PRDX3 in the cardiac tissue of DCM patients probably due to its specific role in the attenuation mechanisms of these failing hearts. Although, we did not observe the same tendency between protein levels and gene expression, likely because of a different removal mechanism of this protein in failing hearts; however, the most remarkable finding was the good correlation between this protein level and LV function, indicating that an increased PRDX3 level is associated with impaired ventricular function. Thus, our findings demonstrated once again that mitochondrial oxidative stress is key player in the pathogenesis of cardiac failure and showed for the first time a direct relationship between the level of this antioxidant and LV dysfunction in human cardiac tissue, suggesting that it could be a primary line of defense against this disease process. With the objective to evaluate the causality of this significant relationship, further studies need to be done.

It is noteworthy that our results are consistent and have been validated by different established techniques and by novel and precise SRM analysis and RNAseq approach. Despite these significant data, more work is needed to fully understand the causal role and which of the alterations observed are adaptive or maladaptive. A promising way to obtain new therapeutic options is to explore strategies based on gene therapy to clarify the mechanism leading to energetic derangement and, moreover, to restore ventricular function in DCM patients. For example, silencing or inducing overexpression of *PRDX3* through gene therapy with the creation of a murine DCM model would allow us to investigate whether LV is aggravated or improved and to develop etiology-specific therapies in HF. In this way, we suggest new molecular targets and all these experiments could be providing a new therapeutic approach.

### Study limitations

A common limitation of studies that use cardiac tissues from end-stage failing human hearts is the fact that there is high variability in disease etiology and treatment. To make our study population etiologically homogeneous, we chose DCM patients who did not report any family history of the disease.

Moreover, our tissue samples were taken from the transmural left ventricle apex, so our findings could not be generalized to all layers and regions of the left ventricle. However, we want to emphasize the importance of having carried out this study in a significant number of samples from explanted human hearts from DCM patients undergoing cardiac transplantation. This has allowed us to extract the region of tissue that we wanted to analyze, which could not have been possible if the study had been made using biopsied tissues.

In summary, the present study is the first to analyze the mitochondrial proteome in cardiac tissue of DCM patients. We found significant and reproducible alterations in cardiac energy metabolism, especially in molecules involved in substrate utilization and energy production, obtaining a considerable relationship between the alterations detected in both processes. LV dysfunction is directly related with the antioxidant PRDX3 expression. This work provides new insight into the cellular mechanisms associated with the pathophysiology of DCM and could serve as a pivotal study to develop etiology-specific therapies in HF.

## Supporting Information

File S1 **Supporting figure and tables.** Figure_S1: Representative two-dimensional DIGE gel. Table_S1: Additional data on MS protein identification of DCM spots with differential expression by MALDI-MS. Table_S2: Additional data on selected reaction monitoring (SRM) analysis. Table_S3: Additional data on selected reaction monitoring (SRM) analysis. Analyte peak area (counts) for each peptide in all samples.
(DOCX)
