# Supplementary material for: Heart Mitochondrial Proteome Study Elucidates Changes in Cardiac Energy Metabolism and Antioxidant PRDX3 in Human Dilated Cardiomyopathy
Source: PLoS One. 2014 Nov 14;9(11):e112971. doi: 10.1371/journal.pone.0112971 (PMC4232587; doi:10.1371/journal.pone.0112971)
Supplement: File S1 — Supporting figure and tables. Figure_S1: Representative two-dimensional DIGE gel. Table_S1: Additional data on MS protein identification of DCM spots with differential expression by MALDI-MS. Table_S2: Additional data on selected reaction monitoring (SRM) analysis. Table_S3: Additional data on selected reaction monitoring (SRM) analysis. Analyte peak area (counts) for each peptide in all samples. (DOCX) [file pone.0112971.s001.docx]

**FILE S1**

**Figure_S1. Representative two-dimensional DIGE gel.** A differential abundance analysis of the dilated hearts proteome was performed by saturation labeling two-dimensional-DIGE analysis. Assayed comparisons were: pathological samples *versus* control. Spots found significantly varied (*p value* < 0.05) and with a fold change greater than 1.5 or lower than -1.5, are represented in a representative two-dimensional DIGE gel (A). Zoom of selected spots (B).

**
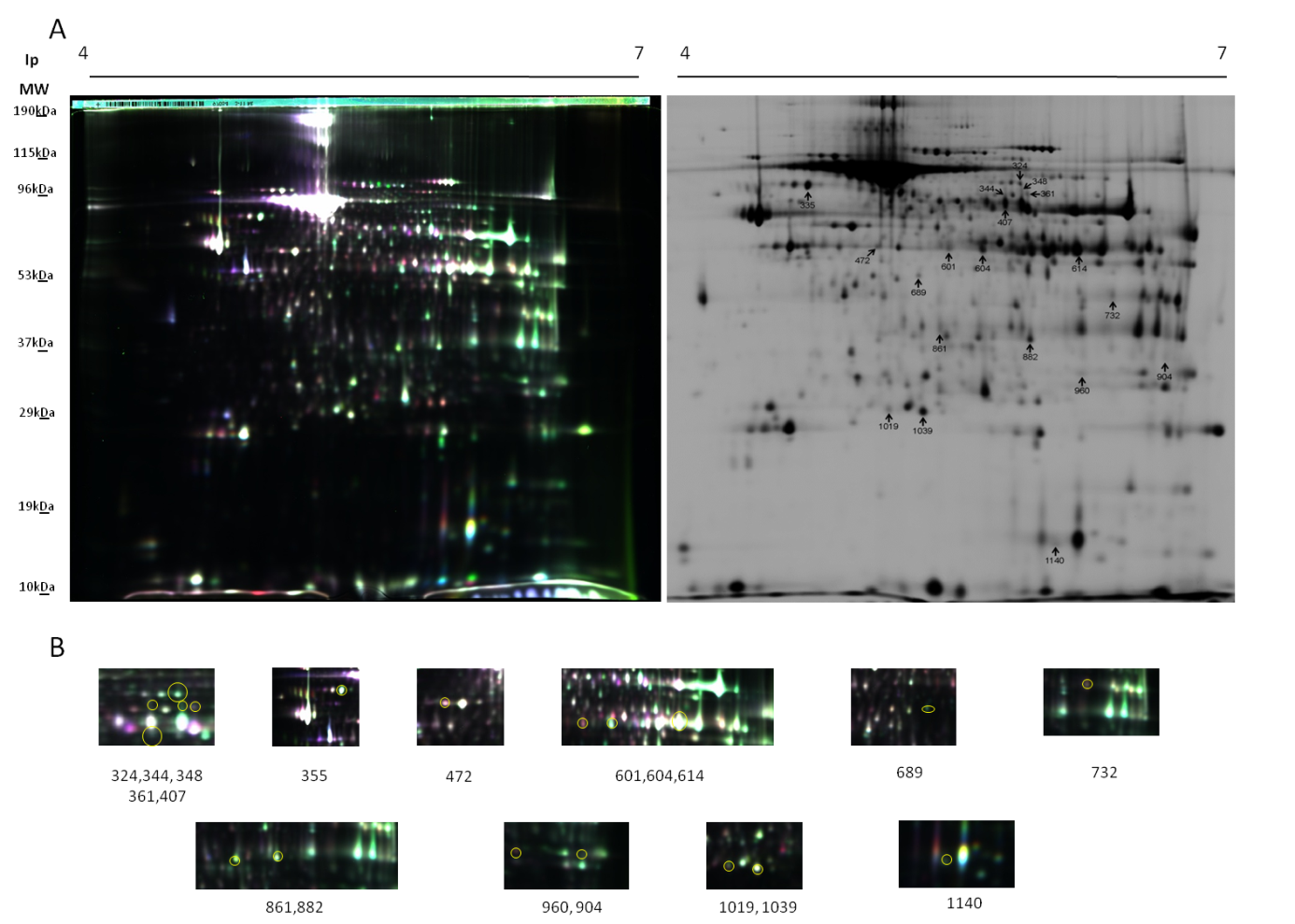
**

**Table_S1:** Additional data on MS protein identification of DCM spots with differential expression by MALDI-MS.

| **S**  **Spot** | **N^a^ / %^b^** | **Mascot Score** | **Peptides identified by MS** | | **Identified protein** | **Accession code** | **Expected Mw** | **Expected pI** |  | |  | |  |
| --- | --- | --- | --- | --- | --- | --- | --- | --- | --- | --- | --- | --- | --- |
|  |  |  | **M+H** | **Sequence** |  |  |  |  | **Experim.**  **Mw** | | **Experim.**  **pI** | |  |
| 324 | 12/29 | 151 | 806.42 | R.FGILTEK.Y | Electron transfer flavoprotein-ubiquinone oxidoreductase, mitochondrial | ETFD_HUMAN | 69250 | 7.31 | 110000 | | 5.90 | |  |
|  |  |  | 818.43 | K.NSWVWK.E |  |  |  |  |  | |  | |  |
|  |  |  | 1030.53 | K.ELWVIDEK.N |  |  |  |  |  | |  | |  |
|  |  |  | 1263.65 | R.ITTHYTIYPR.D |  |  |  |  |  | |  | |  |
|  |  |  | 1290.61 | R.NLSIYDGPEQR.F |  |  |  |  |  | |  | |  |
|  |  |  | 1293.63 | R.VDHTVGWPLDR.H |  |  |  |  |  | |  | |  |
|  |  |  | 1538.75 | K.VTIFAEGCHGHLAK.Q |  |  |  |  |  | |  | |  |
|  |  |  | 2098.09 | R.FAEEADVVIVGAGPAGLSAAVR.L |  |  |  |  |  | |  | |  |
|  |  |  | 2172.99 | R.FCPAGVYEFVPVEQGDGFR.L |  |  |  |  |  | |  | |  |
|  |  |  | 2229.20 | R.IPVPILPGLPMNNHGNYIVR.L + Oxidation (M) |  |  |  |  |  | |  | |  |
|  |  |  | 2249.09 | K.SGILAAESIFNQLTSENLQSK.T |  |  |  |  |  | |  | |  |
|  |  |  | 3345.77 | R.ALNEGGFQSIPKLTFPGGLLIGCSPGFMNVPK.I |  |  |  |  |  | |  | |  |
| 335 | 28/52 | 222 | 832.39 | K.APGFGDNR.K | 60 kDa heat shock protein, mitochondrial | CH60_HUMAN | 61187 | 5.70 | 107000 | | 4.70 | |  |
|  |  |  | 854.47 | K.GANPVEIR.R |  |  |  |  |  | |  | |  |
|  |  |  | 940.61 | K.IGIEIIKR.T |  |  |  |  |  | |  | |  |
|  |  |  | 959.51 | R.VTDALNATR.A |  |  |  |  |  | |  | |  |
|  |  |  | 960.49 | K.APGFGDNRK.N |  |  |  |  |  | |  | |  |
|  |  |  | 1214.65 | K.NAGVEGSLIVEK.I |  |  |  |  |  | |  | |  |
|  |  |  | 1343.71 | R.TVIIEQSWGSPK.V |  |  |  |  |  | |  | |  |
|  |  |  | 1388.70 | R.GYISPYFINTSK.G |  |  |  |  |  | |  | |  |
|  |  |  | 1503.75 | K.TLNDELEIIEGMK.F |  |  |  |  |  | |  | |  |
|  |  |  | 1519.74 | K.TLNDELEIIEGMK.F + Oxidation (M) |  |  |  |  |  | |  | |  |
|  |  |  | 1571.89 | R.GVMLAVDAVIAELKK.Q + Oxidation (M) |  |  |  |  |  | |  | |  |
|  |  |  | 1600.74 | K.CEFQDAYVLLSEK.K |  |  |  |  |  | |  | |  |
|  |  |  | 1645.89 | K.VGEVIVTKDDAMLLK.G + Oxidation (M) |  |  |  |  |  | |  | |  |
|  |  |  | 1683.90 | R.AAVEEGIVLGGGCALLR.C |  |  |  |  |  | |  | |  |
|  |  |  | 1728.84 | K.CEFQDAYVLLSEKK.I |  |  |  |  |  | |  | |  |
|  |  |  | 1770.84 | R.CIPALDSLTPANEDQK.I |  |  |  |  |  | |  | |  |
|  |  |  | 1918.06 | K.ISSIQSIVPALEIANAHR.K |  |  |  |  |  | |  | |  |
|  |  |  | 1937.94 | K.TLNDELEIIEGMKFDR.G + Oxidation (M) |  |  |  |  |  | |  | |  |
|  |  |  | 2037.01 | R.IQEIIEQLDVTTSEYEK.E |  |  |  |  |  | |  | |  |
|  |  |  | 2046.16 | K.KISSIQSIVPALEIANAHR.K |  |  |  |  |  | |  | |  |
|  |  |  | 2128.13 | R.ALMLQGVDLLADAVAVTMGPK.G + Oxidation (M) |  |  |  |  |  | |  | |  |
|  |  |  | 2144.12 | R.ALMLQGVDLLADAVAVTMGPK.G + 2 Oxidation (M) |  |  |  |  |  | |  | |  |
|  |  |  | 2193.11 | K.RIQEIIEQLDVTTSEYEK.E |  |  |  |  |  | |  | |  |
|  |  |  | 2294.16 | R.IQEIIEQLDVTTSEYEKEK.L |  |  |  |  |  | |  | |  |
|  |  |  | 2364.33 | R.KPLVIIAEDVDGEALSTLVLNR.L |  |  |  |  |  | |  | |  |
|  |  |  | 2559.25 | K.LVQDVANNTNEEAGDGTTTATVLAR.S |  |  |  |  |  | |  | |  |
|  |  |  | 3096.57 | K.DMAIATGGAVFGEEGLTLNLEDVQPHDLGK.V |  |  |  |  |  | |  | |  |
|  |  |  | 3112.53 | K.DMAIATGGAVFGEEGLTLNLEDVQPHDLGK.V + Oxid (M) |  |  |  |  |  | |  | |  |
| 344 | 29/52 | 270 | 806.42 | R.FGILTEK.Y | Electron transfer flavoprotein-ubiquinone oxidoreductase, mitochondrial | ETFD_HUMAN | 69250 | 7.31 | 100000 | | 5.60 | |  |
|  |  |  | 818.42 | K.NSWVWK.E |  |  |  |  |  | |  | |  |
|  |  |  | 933.46 | K.ELFPDWK.E |  |  |  |  |  | |  | |  |
|  |  |  | 960.48 | R.GMEPWTLK.H |  |  |  |  |  | |  | |  |
|  |  |  | 1019.45 | R.WEGVNMER.F |  |  |  |  |  | |  | |  |
|  |  |  | 1030.53 | K.ELWVIDEK.N |  |  |  |  |  | |  | |  |
|  |  |  | 1035.45 | R.WEGVNMER.F + Oxidation (M) |  |  |  |  |  | |  | |  |
|  |  |  | 1114.60 | K.GIATNDVGIQK.D |  |  |  |  |  | |  | |  |
|  |  |  | 1190.60 | K.ELFPDWKEK.G |  |  |  |  |  | |  | |  |
|  |  |  | 1259.65 | R.ALNEGGFQSIPK.L |  |  |  |  |  | |  | |  |
|  |  |  | 1263.66 | R.ITTHYTIYPR.D |  |  |  |  |  | |  | |  |
|  |  |  | 1268.63 | K.GAPLNTPVTEDR.F |  |  |  |  |  | |  | |  |
|  |  |  | 1278.70 | K.QLAVAHEKDIR.V |  |  |  |  |  | |  | |  |
|  |  |  | 1290.62 | R.NLSIYDGPEQR.F |  |  |  |  |  | |  | |  |
|  |  |  | 1293.64 | R.VDHTVGWPLDR.H |  |  |  |  |  | |  | |  |
|  |  |  | 1427.77 | K.HHPSIRPTLEGGK.R |  |  |  |  |  | |  | |  |
|  |  |  | 1449.70 | R.ANCEPQTYGIGLK.E |  |  |  |  |  | |  | |  |
|  |  |  | 1483.70 | R.LQINAQNCVHCK.T |  |  |  |  |  | |  | |  |
|  |  |  | 1538.77 | K.VTIFAEGCHGHLAK.Q |  |  |  |  |  | |  | |  |
|  |  |  | 1630.82 | K.TIGLHVTEYEDNLK.N |  |  |  |  |  | |  | |  |
|  |  |  | 1741.90 | R.WKHHPSIRPTLEGGK.R |  |  |  |  |  | |  | |  |
|  |  |  | 1983.98 | K.AAQIGAHTLSGACLDPGAFK.E |  |  |  |  |  | |  | |  |
|  |  |  | 2057.08 | K.GAPLNTPVTEDRFGILTEK.Y |  |  |  |  |  | |  | |  |
|  |  |  | 2098.10 | R.FAEEADVVIVGAGPAGLSAAVR.L |  |  |  |  |  | |  | |  |
|  |  |  | 2120.07 | K.LTFPGGLLIGCSPGFMNVPK.I + Oxidation (M) |  |  |  |  |  | |  | |  |
|  |  |  | 2172.99 | R.FCPAGVYEFVPVEQGDGFR.L |  |  |  |  |  | |  | |  |
|  |  |  | 2229.19 | R.IPVPILPGLPMNNHGNYIVR.L + Oxidation (M) |  |  |  |  |  | |  | |  |
|  |  |  | 2249.14 | K.SGILAAESIFNQLTSENLQSK.T |  |  |  |  |  | |  | |  |
|  |  |  | 2287.08 | R.GLELHAKVTIFAEGCHGHLAK.Q |  |  |  |  |  | |  | |  |
| 348 | 5/13 | 93 | 908.46 | K.FPFAANSR.A | Dihydrolipoyl dehydrogenase, mitochondrial | DLDH_HUMAN | 54713 | 7.95 | 105000 | | 6.00 | |  |
|  |  |  | 1523.73 | R.VCHAHPTLSEAFR.E |  |  |  |  |  | |  | |  |
|  |  |  | 1566.82 | K.NLGLEELGIELDPR.G |  |  |  |  |  | |  | |  |
|  |  |  | 1716.89 | K.AEVITCDVLLVCIGR.R |  |  |  |  |  | |  | |  |
|  |  |  | 1985.02 | K.MVVIGAGVIGVELGSVWQR.L + Oxidation (M) |  |  |  |  |  | |  | |  |
| 361 | 12/14 | 79 | 803.42 | R.FHTFPR.L | Delta-1-pyrroline-5-carboxylate dehydrogenase, mitochondrial | AL4A1_HUMAN | 62137 | 8.28 | 100000 | | 6.10 | |  |
|  |  |  | 882.48 | R.LLEEHSR.I |  |  |  |  |  | |  | |  |
|  |  |  | 955.48 | K.NFHFVHR.S |  |  |  |  |  | |  | |  |
|  |  |  | 985.46 | R.SAFEYGGQK.C |  |  |  |  |  | |  | |  |
|  |  |  | 1225.56 | R.NAAGNFYINDK.S |  |  |  |  |  | |  | |  |
|  |  |  | 1318.67 | R.SADVESVVSGTLR.S |  |  |  |  |  | |  | |  |
|  |  |  | 1460.74 | K.STGSIVGQQPFGGAR.A |  |  |  |  |  | |  | |  |
|  |  |  | 1681.85 | R.ASGTNDKPGGPHYILR.W |  |  |  |  |  | |  | |  |
|  |  |  | 1713.87 | K.VANEPVLAFTQGSPER.D |  |  |  |  |  | |  | |  |
|  |  |  | 1727.86 | K.QVAQNLDRFHTFPR.L |  |  |  |  |  | |  | |  |
|  |  |  | 2269.16 | K.VANEPVLAFTQGSPERDALQK.A |  |  |  |  |  | |  | |  |
|  |  |  | 2534.28 | K.YAVELEGQQPISVPPSTNSTVYR.G |  |  |  |  |  | |  | |  |
| 407 | 17/47 | 114 | 908.45 | K.FPFAANSR.A | Dihydrolipoyl dehydrogenase, mitochondrial | DLDH_HUMAN | 54713 | 7.95 | 90000 | | 5.70 | |  |
|  |  |  | 911.53 | R.GRIPVNTR.F |  |  |  |  |  | |  | |  |
|  |  |  | 1126.65 | K.ALTGGIAHLFK.Q |  |  |  |  |  | |  | |  |
|  |  |  | 1523.73 | R.VCHAHPTLSEAFR.E |  |  |  |  |  | |  | |  |
|  |  |  | 1566.83 | K.NLGLEELGIELDPR.G |  |  |  |  |  | |  | |  |
|  |  |  | 1580.76 | K.SEEQLKEEGIEYK.V |  |  |  |  |  | |  | |  |
|  |  |  | 1716.89 | K.AEVITCDVLLVCIGR.R |  |  |  |  |  | |  | |  |
|  |  |  | 1770.82 | K.ALLNNSHYYHMAHGK.D + Oxidation (M) |  |  |  |  |  | |  | |  |
|  |  |  | 1818.86 | K.NETLGGTCLNVGCIPSK.A |  |  |  |  |  | |  | |  |
|  |  |  | 1969.08 | K.MVVIGAGVIGVELGSVWQR.L |  |  |  |  |  | |  | |  |
|  |  |  | 1978.07 | K.IPNIYAIGDVVAGPMLAHK.A |  |  |  |  |  | |  | |  |
|  |  |  | 1985.08 | K.MVVIGAGVIGVELGSVWQR.L + Oxidation (M) |  |  |  |  |  | |  | |  |
|  |  |  | 1994.06 | K.IPNIYAIGDVVAGPMLAHK.A + Oxidation (M) |  |  |  |  |  | |  | |  |
|  |  |  | 2513.30 | R.LGADVTAVEFLGHVGGVGIDMEISK.N |  |  |  |  |  | |  | |  |
|  |  |  | 2529.30 | R.LGADVTAVEFLGHVGGVGIDMEISK.N + Oxid (M) |  |  |  |  |  | |  | |  |
|  |  |  | 3358.79 | K.NILIATGSEVTPFPGITIDEDTIVSSTGALSLK.K |  |  |  |  |  | |  | |  |
|  |  |  | 3369.68 | R.VLGAHILGPGAGEMVNEAALALEYGASCEDIAR.V + Oxid (M) |  |  |  |  |  | |  | |  |
| 472 | 9/20 | 164 | 834.46 | R.EAVTFLR.K | Dihydrolipoyllysine-residue succinyltransferase component of 2-oxoglutarate dehydrogenase complex, mitochondrial | ODO2_HUMAN | 49067 | 9.11 | 65000 | | 5.10 | |  |
|  |  |  | 851.56 | R.GLVVPVIR.N |  |  |  |  |  | |  | |  |
|  |  |  | 889.48 | R.TITELGEK.A |  |  |  |  |  | |  | |  |
|  |  |  | 1188.65 | K.VEGGTPLFTLR.K |  |  |  |  |  | |  | |  |
|  |  |  | 1407.65 | R.NVEAMNFADIER.T |  |  |  |  |  | |  | |  |
|  |  |  | 1418.73 | R.DYIDISVAVATPR.G |  |  |  |  |  | |  | |  |
|  |  |  | 1423.64 | R.NVEAMNFADIER.T + Oxidation (M) |  |  |  |  |  | |  | |  |
|  |  |  | 1477.71 | K.TPAFAESVTEGDVR.W |  |  |  |  |  | |  | |  |
|  |  |  | 2216.13 | K.ASAFALQEQPVVNAVIDDTTK.E |  |  |  |  |  | |  | |  |
| 601 | 15/31 | 85 | 815.43 | R.GANQWIK.F | Pyruvate dehydrogenase E1 component subunit alpha, somatic form, mitochondrial | ODPA_HUMAN | 43952 | 8.35 | 65000 | | 5.50 | |  |
|  |  |  | 857.39 | R.FAAAYCR.S |  |  |  |  |  | |  | |  |
|  |  |  | 873.47 | R.GDFIPGLR.V |  |  |  |  |  | |  | |  |
|  |  |  | 901.44 | R.EEIQEVR.S |  |  |  |  |  | |  | |  |
|  |  |  | 935.46 | R.AHGFTFTR.G |  |  |  |  |  | |  | |  |
|  |  |  | 1000.56 | R.EILAELTGR.K |  |  |  |  |  | |  | |  |
|  |  |  | 1014.45 | R.YGMGTSVER.A + Oxidation (M) |  |  |  |  |  | |  | |  |
|  |  |  | 1029.57 | K.RGDFIPGLR.V |  |  |  |  |  | |  | |  |
|  |  |  | 1176.57 | R.VDGMDILCVR.E |  |  |  |  |  | |  | |  |
|  |  |  | 1192.57 | R.VDGMDILCVR.E + Oxidation (M) |  |  |  |  |  | |  | |  |
|  |  |  | 1319.69 | K.GPILMELQTYR.Y |  |  |  |  |  | |  | |  |
|  |  |  | 1335.69 | K.GPILMELQTYR.Y + Oxidation (M) |  |  |  |  |  | |  | |  |
|  |  |  | 1410.77 | R.LEEGPPVTTVLTR.E |  |  |  |  |  | |  | |  |
|  |  |  | 1434.67 | K.LPCIFICENNR.Y |  |  |  |  |  | |  | |  |
|  |  |  | 2289.12 | R.MVNSNLASVEELKEIDVEVR.K + Oxidation (M) |  |  |  |  |  | |  | |  |
| 604 | 14/32 | 98 | 821.40 | K.EFQATAR.K | Medium-chain specific acyl-CoA dehydrogenase, mitochondrial | ACADM_HUMAN | 47015 | 8.61 | 65000 | | 5.80 | |  |
|  |  |  | 862.43 | R.CCRVLR.S |  |  |  |  |  | |  | |  |
|  |  |  | 989.46 | R.AAWEVDSGR.R |  |  |  |  |  | |  | |  |
|  |  |  | 1029.51 | R.NTYYASIAK.A |  |  |  |  |  | |  | |  |
|  |  |  | 1143.63 | K.TGEYPVPLIR.R |  |  |  |  |  | |  | |  |
|  |  |  | 1145.56 | R.AAWEVDSGRR.N |  |  |  |  |  | |  | |  |
|  |  |  | 1263.65 | K.KGDEYIINGQK.M |  |  |  |  |  | |  | |  |
|  |  |  | 1375.69 | R.EEIIPVAAEYDK.T |  |  |  |  |  | |  | |  |
|  |  |  | 1464.85 | K.TRPVVAAGAVGLAQR.A |  |  |  |  |  | |  | |  |
|  |  |  | 1597.81 | K.IYQIYEGTSQIQR.L |  |  |  |  |  | |  | |  |
|  |  |  | 1742.82 | R.EPGLGFSFEFTEQQK.E |  |  |  |  |  | |  | |  |
|  |  |  | 1890.98 | K.AFTGFIVEADTPGIQIGR.K |  |  |  |  |  | |  | |  |
|  |  |  | 2026.97 | R.QREPGLGFSFEFTEQQK.E |  |  |  |  |  | |  | |  |
|  |  |  | 2038.98 | K.GDEYIINGQKMWITNGGK.A + Oxidation (M) |  |  |  |  |  | |  | |  |
|  | 14/31 | 85 | 815.43 | R.GANQWIK.F | Pyruvate dehydrogenase E1 component subunit alpha, somatic form, mitochondrial | ODPA_HUMAN | 43952 | 8.35 | | 65000 | | 5.80 | |
|  |  |  | 857.39 | R.FAAAYCR.S |  |  |  |  | |  | |  | |
|  |  |  | 873.47 | R.GDFIPGLR.V |  |  |  |  | |  | |  | |
|  |  |  | 935.46 | R.AHGFTFTR.G |  |  |  |  | |  | |  | |
|  |  |  | 1000.56 | R.EILAELTGR.K |  |  |  |  | |  | |  | |
|  |  |  | 1014.44 | R.YGMGTSVER.A + Oxidation (M) |  |  |  |  | |  | |  | |
|  |  |  | 1029.57 | K.RGDFIPGLR.V |  |  |  |  | |  | |  | |
|  |  |  | 1158.60 | R.TREEIQEVR.S |  |  |  |  | |  | |  | |
|  |  |  | 1192.56 | R.VDGMDILCVR.E + Oxidation (M) |  |  |  |  | |  | |  | |
|  |  |  | 1319.69 | K.GPILMELQTYR.Y |  |  |  |  | |  | |  | |
|  |  |  | 1335.69 | K.GPILMELQTYR.Y + Oxidation (M) |  |  |  |  | |  | |  | |
|  |  |  | 1410.77 | R.LEEGPPVTTVLTR.E |  |  |  |  | |  | |  | |
|  |  |  | 1434.67 | K.LPCIFICENNR.Y |  |  |  |  | |  | |  | |
|  |  |  | 2289.15 | R.MVNSNLASVEELKEIDVEVR.K + Oxidation (M) |  |  |  |  | |  | |  | |
| 614 | 11/28 | 74 | 1070.55 | R.GLSLPPACTR.A | Creatine kinase S-type, mitochondrial | KCRS_HUMAN | 47988 | 8.46 | | 70000 | | 6.30 | |
|  |  |  | 1078.61 | K.VPPPLPQFGK.K |  |  |  |  | |  | |  | |
|  |  |  | 1215.61 | R.HNGYDPRVMK.H |  |  |  |  | |  | |  | |
|  |  |  | 1322.73 | MASIFSKLLTGR.N |  |  |  |  | |  | |  | |
|  |  |  | 1389.70 | R.LFPPSADYPDLR.K |  |  |  |  | |  | |  | |
|  |  |  | 1517.79 | R.LFPPSADYPDLRK.H |  |  |  |  | |  | |  | |
|  |  |  | 1672.82 | K.TFLIWINEEDHTR.V |  |  |  |  | |  | |  | |
|  |  |  | 1733.90 | R.LGYILTCPSNLGTGLR.A |  |  |  |  | |  | |  | |
|  |  |  | 1778.86 | K.ITQGQFDEHYVLSSR.V |  |  |  |  | |  | |  | |
|  |  |  | 2108.00 | R.GTGGVDTAAVADVYDISNIDR.I |  |  |  |  | |  | |  | |
|  |  |  | 2264.05 | K.RGTGGVDTAAVADVYDISNIDR.I |  |  |  |  | |  | |  | |
| 689 | 3/9 | 52 | 814.49 | R.GLAVEAKK.T | Elongation factor Tu, mitochondrial | EFTU_HUMAN | 49852 | 7.26 | | 55000 | | 6.30 | |
|  |  |  | 1541.85 | K.LLDAVDTYIPVPAR.D |  |  |  |  | |  | |  | |
|  |  |  | 2128.16 | R.DLEKPFLLPVEAVYSVPGR.G |  |  |  |  | |  | |  | |
| 732 | 7/28 | 87 | 1152.55 | K.EGVVECSFVK.S | Malate dehydrogenase, mitochondrial | MDHM_HUMAN | 35937 | 8.92 | | 50000 | | 6.50 | |
|  |  |  | 1232.71 | K.IFGVTTLDIVR.A |  |  |  |  | |  | |  | |
|  |  |  | 1337.71 | K.GCDVVVIPAGVPR.K |  |  |  |  | |  | |  | |
|  |  |  | 1342.66 | R.FVFSLVDAMNGK.E + Oxidation (M) |  |  |  |  | |  | |  | |
|  |  |  | 1453.70 | K.AGAGSATLSMAYAGAR.F |  |  |  |  | |  | |  | |
|  |  |  | 1559.79 | K.VDFPQDQLTALTGR.I |  |  |  |  | |  | |  | |
|  |  |  | 2364.23 | R.LTLYDIAHTPGVAADLSHIETK.A |  |  |  |  | |  | |  | |
| 861 | 7/33 | 152 | 909.44 | K.DVCTFLR.W | Cytochrome c1, heme protein, mitochondrial | CY1_HUMAN | 35741 | 9.15 | | 40000 | | 5.20 | |
|  |  |  | 1262.54 | R.WASEPEHDHR.K |  |  |  |  | |  | |  | |
|  |  |  | 1297.64 | R.GLLSSLDHTSIR.R |  |  |  |  | |  | |  | |
|  |  |  | 1669.87 | R.AANNGALPPDLSYIVR.A |  |  |  |  | |  | |  | |
|  |  |  | 1913.92 | K.LFDYFPKPYPNSEAAR.A |  |  |  |  | |  | |  | |
|  |  |  | 2647.21 | K.ELAAEVEVQDGPNEDGEMFMRPGK.L |  |  |  |  | |  | |  | |
|  |  |  | 2653.25 | R.HGGEDYVFSLLTGYCEPPTGVSLR.E |  |  |  |  | |  | |  | |
|  | 6/19 | 147 | 836.46 | R.ISWYLR.D | Delta(3,5)-Delta(2,4)-dienoyl-CoA isomerase, mitochondrial | ECH1_HUMAN | 36136 | 8.16 | | 40000 | | 5.20 | |
|  |  |  | 863.48 | K.VNLLYSR.D |  |  |  |  | |  | |  | |
|  |  |  | 1297.64 | R.YQETFNVIER.C |  |  |  |  | |  | |  | |
|  |  |  | 1375.62 | R.YCAQDAFFQVK.E |  |  |  |  | |  | |  | |
|  |  |  | 1541.80 | K.EVDVGLAADVGTLQR.L |  |  |  |  | |  | |  | |
|  |  |  | 1730.93 | K.VIGNQSLVNELAFTAR.K |  |  |  |  | |  | |  | |
| 882 | 8/20 | 74 | 844.51 | R.STVAQLVK.R | ATP synthase subunit alpha, mitochondrial | ATPA_HUMAN | 59828 | 9.16 | | 38000 | | 6,00 | |
|  |  |  | 891.51 | K.LELAQYR.E |  |  |  |  | |  | |  | |
|  |  |  | 1105.55 | R.NALGSSFIAAR.N |  |  |  |  | |  | |  | |
|  |  |  | 1286.67 | K.HALIIYDDLSK.Q |  |  |  |  | |  | |  | |
|  |  |  | 1552.73 | R.EAYPGDVFYLHSR.L |  |  |  |  | |  | |  | |
|  |  |  | 2229.18 | R.VHGLRNVQAEEMVEFSSGLK.G |  |  |  |  | |  | |  | |
|  |  |  | 2308.14 | K.QGQYSPMAIEEQVAVIYAGVR |  |  |  |  | |  | |  | |
|  |  |  | 2337.14 | R.EVAAFAQFGSDLDAATQQLLSR.G |  |  |  |  | |  | |  | |
| 904 | 7/20 | 107 | 814.4 | R.ELIIGDR.Q | ATP synthase subunit alpha, mitochondrial | ATPA_HUMAN | 59828 | 9.16 | | 36000 | | 6.80 | |
|  |  |  | 875.48 | R.QMSLLLR.R |  |  |  |  | |  | |  | |
|  |  |  | 891.48 | K.LELAQYR.E |  |  |  |  | |  | |  | |
|  |  |  | 1105.59 | R.NALGSSFIAAR.N |  |  |  |  | |  | |  | |
|  |  |  | 1574.77 | R.ILGADTSVDLEETGR.V |  |  |  |  | |  | |  | |
|  |  |  | 1623.88 | R.TGAIVDVPVGEELLGR.V |  |  |  |  | |  | |  | |
|  |  |  | 1666.80 | R.NVQAEEMVEFSSGLK.G |  |  |  |  | |  | |  | |
| 960 | 12/33 | 125 | 816.41 | K.ELDRER.A | Coiled-coil-helix-coiled-coil-helix domain-containing protein 3, mitochondrial | CHCH3_HUMAN | 26421 | 8.48 | | 35000 | | 6.30 | |
|  |  |  | 851.43 | K.ILQCYR.E |  |  |  |  | |  | |  | |
|  |  |  | 972.50 | R.AAANEQLTR.A |  |  |  |  | |  | |  | |
|  |  |  | 1199.64 | R.VAEELALEQAK.K |  |  |  |  | |  | |  | |
|  |  |  | 1516.70 | R.YESHPVCADLQAK.I |  |  |  |  | |  | |  | |
|  |  |  | 1574.71 | R.YSGAYGASVSDEELK.R |  |  |  |  | |  | |  | |
|  |  |  | 1706.84 | R.VTFEADENENITVVK.G |  |  |  |  | |  | |  | |
|  |  |  | 1862.94 | R.RVTFEADENENITVVK.G |  |  |  |  | |  | |  | |
| 1019 | 7/42 | 145 | 832.44 | R.SVEETLR.L | Thioredoxin-dependent peroxide reductase, mitochondrial oxoglutarate dehydrogenase complex, mitochondrial | PRDX3_HUMAN | 28017 | 7.67 | | 31000 | | 5.10 | |
|  |  |  | 1205.65 | K.HLSVNDLPVGR.S |  |  |  |  | |  | |  | |
|  |  |  | 1284.74 | R.GLFIIDPNGVIK.H |  |  |  |  | |  | |  | |
|  |  |  | 1461.78 | R.DYGVLLEGSGLALR.G |  |  |  |  | |  | |  | |
|  |  |  | 1565.73 | R.TSLTNLLCSGSSQAK.L |  |  |  |  | |  | |  | |
|  |  |  | 1953.94 | K.GTAVVNGEFKDLSLDDFK.G |  |  |  |  | |  | |  | |
|  |  |  | 3384.64 | K.AFQYVETHGEVCPANWTPDSPTIKPSPAASK.E |  |  |  |  | |  | |  | |
|  | 6/33 | 80 | 1237.68 | K.VAEVLQVPPMR.V | NADH dehydrogenase [ubiquinone] flavoprotein 2, mitochondrial | NDUV2_HUMN | 27659 | 8.22 | | 31000 | | 5.10 | |
|  |  |  | 1253.67 | K.VAEVLQVPPMR.V + Oxidation (M) |  |  |  |  | |  | |  | |
|  |  |  | 1335.79 | K.AAAVLPVLDLAQR.Q |  |  |  |  | |  | |  | |
|  |  |  | 1671.76 | R.VYEVATFYTMYNR.K + Oxidation (M) |  |  |  |  | |  | |  | |
|  |  |  | 2396.05 | R.DTPENNPDTPFDFTPENYKR.I |  |  |  |  | |  | |  | |
|  |  |  | 2673.31 | R.FSCEPAGGLTSLTEPPKGPGFGVQAGL |  |  |  |  | |  | |  | |
| 1039 | 10/43 | 78 | 832.43 | R.SVEETLR.L | Thioredoxin-dependent peroxide reductase, mitochondrial | PRDX3_HUMAN | 28017 | 7.67 | | 30000 | | 5.30 | |
|  |  |  | 1020.52 | K.GTAVVNGEFK.D |  |  |  |  | |  | |  | |
|  |  |  | 1136.57 | K.DLSLDDFKGK.Y |  |  |  |  | |  | |  | |
|  |  |  | 1205.65 | K.HLSVNDLPVGR.S |  |  |  |  | |  | |  | |
|  |  |  | 1284.74 | R.GLFIIDPNGVIK.H |  |  |  |  | |  | |  | |
|  |  |  | 1461.78 | R.DYGVLLEGSGLALR.G |  |  |  |  | |  | |  | |
|  |  |  | 1752.92 | K.NGGLGHMNIALLSDLTK.Q |  |  |  |  | |  | |  | |
|  |  |  | 1768.91 | K.NGGLGHMNIALLSDLTK.Q + Oxidation (M) |  |  |  |  | |  | |  | |
|  |  |  | 1953.97 | K.GTAVVNGEFKDLSLDDFK.G |  |  |  |  | |  | |  | |
|  |  |  | 3384.61 | K.AFQYVETHGEVCPANWTPDSPTIKPSPAASK.E |  |  |  |  | |  | |  | |
| 1140 | 7/46 | 222 | 932.53 | K.VNLAELFK.G | Peroxiredoxin-5, mitochondrial | PRDX5_HUMAN | 22301 | 8.93 | | 15000 | | 6.20 | |
|  |  |  | 1088.59 | R.LLADPTGAFGK.E |  |  |  |  | |  | |  | |
|  |  |  | 1237.64 | R.FSMVVQDGIVK.A + Oxidation (M) |  |  |  |  | |  | |  | |
|  |  |  | 1538.81 | K.THLPGFVEQAEALK.A |  |  |  |  | |  | |  | |
|  |  |  | 1826.91 | K.VGDAIPAVEVFEGEPGNK.V |  |  |  |  | |  | |  | |
|  |  |  | 1905.97 | K.ETDLLLDDSLVSIFGNR.R |  |  |  |  | |  | |  | |
|  |  |  | 2263.11 | K.GVQVVACLSVNDAFVTGEWGR.A |  |  |  |  | |  | |  | |

Detected ion charge: +1 charge

**Table_S2:** Additional data on selected reaction monitoring (SRM) analysis.

| Accession code | Accession nº | Peptide Sequence | Q1 | Q3 | CE | Dwell time | Charge state | Fragment Ion |
| --- | --- | --- | --- | --- | --- | --- | --- | --- |
| ATPA_HUMAN | P25705 | AVDSLVPIGR | 513.80 | 541.35 | 27.61 | 20 | +2 | y5 |
| ATPA_HUMAN | P25705 | AVDSLVPIGR | 513.80 | 654.43 | 28.00 | 20 | +2 | y6 |
| ATPA_HUMAN | P25705 | AVDSLVPIGR | 513.80 | 856.49 | 28.00 | 20 | +2 | y8 |
| ATPA_HUMAN | P25705 | TGAIVDVPVGEELLGR | 812.95 | 969.54 | 41.00 | 20 | +2 | y9 |
| ATPA_HUMAN | P25705 | TGAIVDVPVGEELLGR | 812.95 | 1068.60 | 41.00 | 20 | +2 | y10 |
| ATPA_HUMAN | P25705 | TGAIVDVPVGEELLGR | 812.95 | 1183.63 | 40.77 | 20 | +2 | y11 |
| AL4A1_HUMAN | P30038 | C[CAM]DDSVGYFVEPC[CAM]IVESK | 668.63 | 462.26 | 37.43 | 50 | +3 | y4 |
| AL4A1_HUMAN | P30038 | C[CAM]DDSVGYFVEPC[CAM]IVESK | 668.63 | 832.42 | 37.00 | 50 | +3 | y7 |
| AL4A1_HUMAN | P30038 | C[CAM]DDSVGYFVEPC[CAM]IVESK | 668.63 | 961.47 | 37.00 | 50 | +3 | y8 |
| AL4A1_HUMAN | P30038 | STGSIVGQQPFGGAR | 731.38 | 604.32 | 37.18 | 50 | +2 | y6 |
| AL4A1_HUMAN | P30038 | STGSIVGQQPFGGAR | 731.38 | 917.46 | 37.00 | 50 | +2 | y9 |
| AL4A1_HUMAN | P30038 | STGSIVGQQPFGGAR | 731.38 | 1016.53 | 37.00 | 50 | +2 | y10 |
| DLDH_HUMAN | P09622 | ISHGLQGLSAVPLR | 724.42 | 642.39 | 36.87 | 20 | +2 | y6 |
| DLDH_HUMAN | P09622 | ISHGLQGLSAVPLR | 724.42 | 812.50 | 37.00 | 20 | +2 | y8 |
| DLDH_HUMAN | P09622 | ISHGLQGLSAVPLR | 724.42 | 940.56 | 37.00 | 20 | +2 | y9 |
| DLDH_HUMAN | P09622 | NLGLEELGIELDPR | 784.42 | 799.43 | 39.51 | 20 | +2 | y7 |
| DLDH_HUMAN | P09622 | NLGLEELGIELDPR | 784.42 | 1041.56 | 40.00 | 20 | +2 | y9 |
| DLDH_HUMAN | P09622 | NLGLEELGIELDPR | 784.42 | 1170.60 | 39.51 | 20 | +2 | y10 |
| ETFD_HUMAN | Q16134 | ALNEGGFQSIPK | 630.83 | 833.45 | 33.00 | 20 | +2 | y8 |
| ETFD_HUMAN | Q16134 | ALNEGGFQSIPK | 630.83 | 962.49 | 33.00 | 20 | +2 | y9 |
| ETFD_HUMAN | Q16134 | ALNEGGFQSIPK | 630.83 | 1076.54 | 32.76 | 20 | +2 | y10 |
| ETFD_HUMAN | Q16134 | NLSIYDGPEQR | 646.32 | 586.29 | 33.44 | 20 | +2 | y5 |
| ETFD_HUMAN | Q16134 | NLSIYDGPEQR | 646.32 | 864.38 | 33.00 | 20 | +2 | y7 |
| ETFD_HUMAN | Q16134 | NLSIYDGPEQR | 646.32 | 1064.50 | 33.00 | 20 | +2 | y9 |
| EFTU_HUMAN | P49411 | AEAGDNLGALVR | 593.31 | 515.33 | 31.11 | 20 | +2 | y5 |
| EFTU_HUMAN | P49411 | AEAGDNLGALVR | 593.31 | 742.46 | 31.00 | 20 | +2 | y7 |
| EFTU_HUMAN | P49411 | AEAGDNLGALVR | 593.31 | 985.54 | 31.00 | 20 | +2 | y10 |
| EFTU_HUMAN | P49411 | LLDAVDTYIPVPAR | 771.93 | 539.33 | 38.96 | 20 | +2 | y5 |
| EFTU_HUMAN | P49411 | LLDAVDTYIPVPAR | 771.93 | 652.41 | 38.96 | 20 | +2 | y6 |
| EFTU_HUMAN | P49411 | LLDAVDTYIPVPAR | 771.93 | 1031.55 | 39.00 | 20 | +2 | y9 |
| ODPA_HUMAN | P08559 | EILAELTGR | 501.28 | 575.31 | 27.06 | 20 | +2 | y5 |
| ODPA_HUMAN | P08559 | EILAELTGR | 501.28 | 646.35 | 27.00 | 20 | +2 | y6 |
| ODPA_HUMAN | P08559 | EILAELTGR | 501.28 | 759.44 | 27.00 | 20 | +2 | y7 |
| ODPA_HUMAN | P08559 | LPC[CAM]IFIC[CAM]ENNR | 718.34 | 692.28 | 36.61 | 20 | +2 | y5 |
| ODPA_HUMAN | P08559 | LPC[CAM]IFIC[CAM]ENNR | 718.34 | 805.36 | 36.61 | 20 | +2 | y6 |
| ODPA_HUMAN | P08559 | LPC[CAM]IFIC[CAM]ENNR | 718.34 | 952.43 | 37.00 | 20 | +2 | y7 |
| PRDX3_HUMAN | P30048 | GLFIIDPNGVIK | 643.38 | 627.38 | 33.31 | 20 | +2 | y6 |
| PRDX3_HUMAN | P30048 | GLFIIDPNGVIK | 643.38 | 742.41 | 33.31 | 20 | +2 | y7 |
| PRDX3_HUMAN | P30048 | GLFIIDPNGVIK | 643.38 | 855.49 | 33.00 | 20 | +2 | y8 |
| PRDX3_HUMAN | P30048 | DYGVLLEGSGLALR | 731.90 | 673.40 | 37.20 | 20 | +2 | y7 |
| PRDX3_HUMAN | P30048 | DYGVLLEGSGLALR | 731.90 | 915.53 | 37.00 | 20 | +2 | y9 |
| PRDX3_HUMAN | P30048 | DYGVLLEGSGLALR | 731.90 | 1028.61 | 37.00 | 20 | +2 | y10 |

**Table_S3:** Additional data on selected reaction monitoring (SRM) analysis. Analyte peak area (counts) for each peptide in all samples (four samples per pool).

|  | POOL 1 DCM | POOL 2 DCM | POOL 3 DCM | POOL 4 DCM | POOL 5 CNT | POOL 6 CNT |
| --- | --- | --- | --- | --- | --- | --- |
|  |  |  |  |  |  |  |
| ODPA_EILAELTGR.2y5 | 1,77E+05 | 1,46E+05 | 1,37E+05 | 9,06E+04 | 2,86E+04 | 1,72E+04 |
|  | 1,56E+05 | 1,39E+05 | 9,19E+04 | 2,41E+04 | 2,55E+04 | 1,67E+04 |
|  | 1,47E+05 | 1,23E+05 | 1,12E+05 | 9,00E+04 | 2,45E+04 | 1,58E+04 |
|  |  |  |  |  |  |  |
| ODPA_EILAELTGR.2y6 | 1,83E+05 | 1,44E+05 | 1,32E+05 | 1,22E+05 | 2,99E+04 | 1,84E+04 |
|  | 1,60E+05 | 1,11E+05 | 1,23E+05 | 5,45E+03 | 2,64E+04 | 1,77E+04 |
|  | 1,60E+05 | 1,11E+05 | 1,17E+05 | 9,80E+04 | 2,61E+04 | 1,71E+04 |
|  |  |  |  |  |  |  |
| ODPA_EILAELTGR.2y7 | 1,15E+05 | 1,01E+05 | 9,19E+04 | 6,43E+04 | 2,02E+04 | 1,20E+04 |
|  | 1,10E+05 | 8,64E+04 | 8,37E+04 | 3,23E+03 | 1,71E+04 | 1,15E+04 |
|  | 1,07E+05 | 7,89E+04 | 7,67E+04 | 6,77E+04 | 1,62E+04 | 1,08E+04 |
|  |  |  |  |  |  |  |
| ETFD_ALNEGGFQSIPK.2y8 | 1,78E+04 | 1,51E+04 | 7,57E+04 | 2,36E+04 | 8,64E+03 | 4,90E+03 |
|  | 2,96E+04 | 1,74E+04 | 9,10E+04 | 6,84E+04 | 8,46E+03 | 4,60E+03 |
|  | 3,12E+04 | 2,12E+04 | 9,98E+04 | 5,23E+04 | 8,14E+03 | 4,54E+03 |
|  |  |  |  |  |  |  |
| ETFD_ALNEGGFQSIPK.2y9 | 5,52E+03 | 4,09E+03 | 3,45E+03 | 1,11E+03 | 2,84E+03 | 1,47E+03 |
|  | 8,72E+03 | 7,52E+03 | 3,88E+03 | 7,85E+03 | 2,67E+03 | 1,50E+03 |
|  | 7,83E+03 | 7,32E+03 | 3,37E+03 | 1,30E+03 | 2,27E+03 | 1,44E+03 |
|  |  |  |  |  |  |  |
| ETFD_ALNEGGFQSIPK.2y10 | 8,53E+04 | 7,28E+04 | 3,08E+04 | 6,40E+04 | 4,25E+04 | 2,27E+04 |
|  | 1,35E+04 | 7,67E+04 | 3,55E+04 | 2,74E+04 | 3,98E+04 | 2,14E+04 |
|  | 1,17E+04 | 8,91E+04 | 2,81E+04 | 1,30E+04 | 3,25E+04 | 2,11E+04 |
|  |  |  |  |  |  |  |
| DLDH_ISHGLQGLSAVPLR.2y6 | 1,01E+05 | 6,42E+04 | 7,90E+04 | 9,13E+04 | 7,73E+04 | 5,85E+04 |
|  | 9,54E+04 | 6,90E+04 | 6,39E+04 | 8,23E+04 | 6,49E+04 | 6,23E+04 |
|  | 8,73E+04 | 6,47E+04 | 7,20E+04 | 6,07E+04 | 5,20E+04 | 5,01E+04 |
|  |  |  |  |  |  |  |
| DLDH_ISHGLQGLSAVPLR.2y8 | 4,87E+04 | 3,32E+04 | 3,80E+04 | 4,44E+04 | 3,44E+04 | 2,99E+04 |
|  | 3,69E+04 | 3,10E+04 | 3,69E+04 | 3,30E+04 | 2,81E+04 | 2,25E+04 |
|  | 4,25E+04 | 3,65E+04 | 3,72E+04 | 3,26E+04 | 3,19E+04 | 2,28E+04 |
|  |  |  |  |  |  |  |
| DLDH_ISHGLQGLSAVPLR.2y9 | 5,27E+04 | 3,62E+04 | 4,74E+04 | 4,39E+04 | 4,45E+04 | 3,02E+04 |
|  | 4,93E+04 | 4,06E+04 | 4,43E+04 | 4,45E+04 | 3,83E+04 | 3,38E+04 |
|  | 4,46E+04 | 4,93E+04 | 4,28E+04 | 3,79E+04 | 3,95E+04 | 3,12E+04 |
|  |  |  |  |  |  |  |
| AL4A1_C[CAM]DDSVGYFVEPC[CAM]IVESK.3y4 | 7,38E+03 | 2,01E+03 | 2,04E+03 | 1,29E+03 | 1,05E+03 | 1,58E+03 |
|  | 7,17E+03 | 3,82E+03 | 7,97E+03 | 8,09E+04 | 9,55E+02 | 1,74E+03 |
|  | 7,88E+03 | 4,52E+03 | 1,50E+03 | 1,69E+03 | 1,35E+03 | 1,79E+03 |
|  |  |  |  |  |  |  |
| AL4A1_C[CAM]DDSVGYFVEPC[CAM]IVESK.3y7 | 9,15E+03 | 4,32E+03 | 2,92E+03 | 2,06E+03 | 1,70E+03 | 3,59E+03 |
|  | 9,87E+03 | 4,72E+03 | 3,91E+03 | 1,08E+04 | 2,23E+03 | 3,79E+03 |
|  | 1,04E+04 | 9,23E+03 | 3,26E+03 | 4,60E+03 | 1,86E+03 | 3,43E+03 |
|  |  |  |  |  |  |  |
| AL4A1_C[CAM]DDSVGYFVEPC[CAM]IVESK.3y8 | 4,51E+03 | 1,39E+03 | 8,94E+04 | 2,64E+03 | 8,11E+03 | 1,18E+03 |
|  | 3,74E+03 | 1,62E+03 | 8,20E+04 | 2,08E+04 | 9,72E+03 | 1,49E+03 |
|  | 4,29E+03 | 4,17E+03 | 8,88E+04 | 3,14E+03 | 7,49E+03 | 1,07E+03 |
|  |  |  |  |  |  |  |
| ATPA_AVDSLVPIGR.2y5 | 1,15E+05 | 1,23E+05 | 1,13E+05 | 4,73E+04 | 3,14E+04 | 2,07E+04 |
|  | 1,20E+05 | 1,20E+05 | 1,07E+05 | 3,31E+04 | 3,12E+04 | 2,03E+04 |
|  | 1,69E+05 | 3,93E+05 | 1,04E+05 | 6,85E+04 | 3,05E+04 | 1,94E+04 |
|  |  |  |  |  |  |  |
| ATPA_AVDSLVPIGR.2y6 | 3,03E+04 | 2,77E+04 | 2,39E+04 | 8,63E+03 | 7,54E+03 | 4,92E+03 |
|  | 2,80E+04 | 3,41E+04 | 2,60E+04 | 9,27E+03 | 7,52E+03 | 4,88E+03 |
|  | 4,52E+04 | 9,95E+04 | 2,16E+04 | 1,68E+04 | 6,94E+03 | 4,60E+03 |
|  |  |  |  |  |  |  |
| ATPA_AVDSLVPIGR.2y8 | 6,89E+04 | 7,79E+04 | 7,31E+04 | 2,74E+04 | 2,10E+04 | 1,35E+04 |
|  | 7,82E+04 | 8,25E+04 | 7,15E+04 | 1,31E+04 | 2,09E+04 | 1,36E+04 |
|  | 1,11E+05 | 2,61E+05 | 6,96E+04 | 3,85E+04 | 1,93E+04 | 1,28E+04 |
|  |  |  |  |  |  |  |
| EFTU_AEAGDNLGALVR.2y5 | 1,31E+04 | 1,15E+04 | 1,30E+04 | 8,44E+03 | 2,30E+03 | 1,55E+03 |
|  | 1,65E+04 | 7,45E+03 | 1,99E+04 | 5,45E+03 | 2,28E+03 | 1,77E+03 |
|  | 1,51E+04 | 3,32E+04 | 1,55E+04 | 1,05E+04 | 1,91E+03 | 1,57E+03 |
|  |  |  |  |  |  |  |
| EFTU_AEAGDNLGALVR.2y7 | 5,47E+03 | 6,17E+03 | 5,06E+03 | 2,41E+03 | 3,64E+03 | 3,20E+03 |
|  | 5,35E+03 | 4,21E+03 | 6,11E+03 | 2,08E+03 | 3,89E+03 | 2,92E+03 |
|  | 3,18E+03 | 5,52E+03 | 4,52E+03 | 3,30E+03 | 3,66E+03 | 2,47E+03 |
|  |  |  |  |  |  |  |
| EFTU_AEAGDNLGALVR.2y10 | 6,09E+04 | 4,41E+04 | 7,13E+03 | 2,15E+03 | 1,02E+03 | 7,41E+03 |
|  | 5,22E+04 | 4,46E+04 | 5,68E+03 | 9,69E+03 | 9,74E+03 | 7,42E+03 |
|  | 5,81E+04 | 1,93E+04 | 5,94E+03 | 3,03E+03 | 9,94E+03 | 6,76E+03 |
|  |  |  |  |  |  |  |
| PRDX3_GLFIIDPNGVIK.2y6 | 2,90E+05 | 2,51E+05 | 2,17E+05 | 2,59E+05 | 4,09E+04 | 2,48E+05 |
|  | 2,83E+05 | 2,46E+05 | 2,10E+05 | 2,43E+05 | 3,67E+04 | 2,40E+05 |
|  | 2,85E+05 | 2,60E+05 | 1,85E+05 | 2,22E+05 | 4,23E+04 | 2,21E+05 |
|  |  |  |  |  |  |  |
| PRDX3_GLFIIDPNGVIK.2y7 | 1,12E+05 | 1,09E+05 | 9,06E+04 | 1,05E+05 | 1,74E+04 | 9,76E+04 |
|  | 1,15E+05 | 1,05E+05 | 8,25E+04 | 1,03E+05 | 1,54E+04 | 1,00E+04 |
|  | 1,11E+05 | 1,07E+05 | 7,96E+04 | 9,42E+04 | 1,46E+04 | 8,78E+04 |
|  |  |  |  |  |  |  |
| PRDX3_GLFIIDPNGVIK.2y8 | 1,87E+05 | 1,31E+05 | 1,40E+05 | 1,65E+05 | 2,54E+04 | 1,42E+05 |
|  | 1,56E+05 | 1,41E+05 | 1,18E+05 | 1,38E+05 | 2,02E+04 | 1,40E+05 |
|  | 1,57E+05 | 1,56E+05 | 1,02E+05 | 1,32E+05 | 2,10E+04 | 1,27E+05 |
|  |  |  |  |  |  |  |
| ODPA_LPC[CAM]IFIC[CAM]ENNR.2y5 | 6,54E+04 | 4,52E+04 | 4,03E+04 | 6,19E+04 | 6,54E+03 | 3,34E+04 |
|  | 6,35E+04 | 4,64E+04 | 4,12E+04 | 3,99E+04 | 4,94E+03 | 4,17E+04 |
|  | 6,41E+04 | 4,06E+04 | 4,16E+04 | 4,12E+04 | 5,39E+03 | 3,99E+04 |
|  |  |  |  |  |  |  |
| ODPA_LPC[CAM]IFIC[CAM]ENNR.2y6 | 5,70E+04 | 4,59E+04 | 3,79E+04 | 5,37E+04 | 6,53E+03 | 3,89E+04 |
|  | 6,14E+04 | 4,13E+04 | 3,64E+04 | 3,89E+04 | 5,26E+03 | 4,00E+04 |
|  | 5,42E+04 | 3,95E+04 | 3,80E+04 | 3,84E+04 | 4,66E+03 | 3,52E+04 |
|  |  |  |  |  |  |  |
| ODPA_LPC[CAM]IFIC[CAM]ENNR.2y7 | 1,26E+05 | 1,04E+05 | 8,39E+04 | 9,91E+04 | 1,15E+04 | 7,51E+04 |
|  | 1,05E+05 | 8,75E+04 | 7,02E+04 | 6,61E+04 | 1,19E+04 | 6,72E+04 |
|  | 1,17E+05 | 7,73E+04 | 6,84E+04 | 7,07E+04 | 1,13E+04 | 6,66E+04 |
|  |  |  |  |  |  |  |
| ETFD_NLSIYDGPEQR.2y5 | 3,04E+04 | 2,22E+04 | 2,55E+04 | 2,41E+04 | 6,88E+03 | 6,49E+03 |
|  | 2,98E+04 | 2,36E+04 | 2,73E+04 | 2,20E+04 | 8,60E+03 | 6,59E+03 |
|  | 3,15E+04 | 2,88E+04 | 3,00E+04 | 2,34E+04 | 7,45E+03 | 6,43E+03 |
|  |  |  |  |  |  |  |
| ETFD_NLSIYDGPEQR.2y7 | 3,78E+03 | 2,59E+03 | 2,18E+03 | 8,66E+04 | 3,43E+03 | 3,67E+03 |
|  | 3,30E+03 | 2,68E+03 | 2,13E+03 | 1,07E+04 | 5,40E+03 | 3,90E+03 |
|  | 4,26E+03 | 3,06E+03 | 3,39E+03 | 2,00E+04 | 4,19E+03 | 3,49E+03 |
|  |  |  |  |  |  |  |
| ETFD_NLSIYDGPEQR.2y9 | 3,21E+03 | 2,55E+03 | 2,96E+04 | 1,12E+04 | 3,75E+03 | 3,55E+03 |
|  | 2,99E+03 | 2,88E+03 | 3,27E+04 | 2,39E+04 | 5,51E+03 | 4,24E+03 |
|  | 4,56E+03 | 4,34E+03 | 1,78E+04 | 2,47E+04 | 4,20E+03 | 3,64E+03 |
|  |  |  |  |  |  |  |
| DLDH_NLGLEELGIELDPR.2y7 | 2,54E+05 | 2,21E+05 | 2,33E+05 | 2,64E+05 | 2,15E+05 | 1,12E+05 |
|  | 2,26E+05 | 2,23E+05 | 2,24E+05 | 2,35E+05 | 1,92E+05 | 1,08E+05 |
|  | 2,26E+05 | 1,69E+05 | 2,11E+05 | 2,00E+05 | 1,81E+05 | 9,33E+04 |
|  |  |  |  |  |  |  |
| DLDH_NLGLEELGIELDPR.2y9 | 8,75E+04 | 8,28E+04 | 8,00E+04 | 9,88E+04 | 7,29E+04 | 3,98E+04 |
|  | 8,01E+04 | 7,92E+04 | 8,36E+04 | 8,80E+04 | 6,68E+04 | 3,98E+04 |
|  | 8,25E+04 | 5,92E+04 | 7,55E+04 | 6,49E+04 | 6,53E+04 | 3,59E+04 |
|  |  |  |  |  |  |  |
| DLDH_NLGLEELGIELDPR.2y10 | 1,26E+05 | 9,99E+04 | 1,23E+05 | 1,40E+05 | 1,00E+05 | 5,22E+04 |
|  | 1,20E+05 | 1,18E+05 | 1,14E+05 | 1,22E+05 | 9,15E+04 | 5,33E+04 |
|  | 1,14E+05 | 8,46E+04 | 1,06E+05 | 9,96E+04 | 9,36E+04 | 5,31E+04 |
|  |  |  |  |  |  |  |
| AL4A1_STGSIVGQQPFGGAR.2y6 | 3,08E+03 | 2,89E+03 | 2,70E+03 | 1,93E+03 | 1,21E+03 | 1,01E+03 |
|  | 4,63E+03 | 4,41E+03 | 2,37E+03 | 2,32E+03 | 1,58E+03 | 1,81E+03 |
|  | 2,29E+03 | 6,08E+03 | 2,13E+03 | 2,08E+03 | 1,56E+03 | 1,22E+03 |
|  |  |  |  |  |  |  |
| AL4A1_STGSIVGQQPFGGAR.2y9 | 2,59E+03 | 2,70E+03 | 2,59E+03 | 1,06E+03 | 1,55E+03 | 1,33E+03 |
|  | 2,70E+03 | 2,29E+03 | 2,70E+03 | 1,56E+03 | 1,95E+03 | 1,37E+03 |
|  | 3,04E+03 | 5,33E+03 | 2,03E+03 | 2,49E+03 | 1,64E+03 | 1,44E+03 |
|  |  |  |  |  |  |  |
| AL4A1_STGSIVGQQPFGGAR.2y10 | 1,98E+03 | 1,64E+03 | 2,18E+03 | 1,58E+03 | 1,25E+03 | 1,05E+03 |
|  | 2,25E+03 | 2,45E+03 | 1,47E+03 | 1,87E+03 | 1,50E+03 | 1,41E+03 |
|  | 1,96E+03 | 4,67E+03 | 2,89E+03 | 2,73E+03 | 1,22E+03 | 1,67E+03 |
|  |  |  |  |  |  |  |
| ATPA_TGAIVDVPVGEELLGR.2y9 | 6,83E+06 | 5,05E+06 | 4,88E+06 | 5,19E+06 | 5,57E+05 | 3,43E+06 |
|  | 5,43E+06 | 5,97E+06 | 5,06E+06 | 5,25E+06 | 6,09E+05 | 3,22E+06 |
|  | 5,41E+06 | 4,48E+06 | 4,30E+06 | 4,75E+06 | 6,04E+05 | 3,05E+06 |
|  |  |  |  |  |  |  |
| ATPA_TGAIVDVPVGEELLGR.2y10 | 1,02E+06 | 7,57E+05 | 6,50E+05 | 7,48E+05 | 8,68E+04 | 4,80E+05 |
|  | 9,33E+05 | 8,24E+05 | 7,03E+05 | 7,25E+05 | 8,83E+04 | 4,65E+05 |
|  | 7,78E+05 | 6,47E+05 | 6,01E+05 | 5,69E+05 | 7,42E+04 | 4,32E+05 |
|  |  |  |  |  |  |  |
| ATPA_TGAIVDVPVGEELLGR.2y11 | 2,32E+06 | 2,17E+06 | 2,16E+06 | 2,03E+06 | 2,47E+05 | 1,25E+06 |
|  | 2,14E+06 | 2,11E+06 | 1,91E+06 | 1,97E+06 | 2,36E+05 | 1,21E+06 |
|  | 2,05E+06 | 1,50E+06 | 1,85E+06 | 1,51E+06 | 2,07E+05 | 1,18E+06 |
|  |  |  |  |  |  |  |
| EFTU_LLDAVDTYIPVPAR.2y5 | 3,90E+05 | 2,26E+05 | 3,47E+05 | 4,29E+05 | 2,96E+05 | 1,83E+05 |
|  | 3,44E+05 | 2,66E+05 | 3,31E+05 | 3,21E+05 | 2,65E+05 | 1,73E+05 |
|  | 3,03E+05 | 2,53E+05 | 3,03E+05 | 3,13E+05 | 2,64E+05 | 1,71E+05 |
|  |  |  |  |  |  |  |
| EFTU_LLDAVDTYIPVPAR.2y6 | 1,23E+05 | 7,00E+04 | 1,06E+05 | 1,18E+05 | 6,93E+04 | 6,26E+04 |
|  | 1,07E+05 | 7,36E+04 | 1,00E+05 | 1,08E+05 | 8,50E+04 | 5,43E+04 |
|  | 1,06E+05 | 7,26E+04 | 9,74E+04 | 8,85E+04 | 8,57E+04 | 5,33E+04 |
|  |  |  |  |  |  |  |
| EFTU_LLDAVDTYIPVPAR.2y9 | 1,92E+05 | 1,38E+05 | 2,04E+05 | 2,10E+05 | 1,40E+05 | 9,00E+04 |
|  | 1,44E+05 | 1,23E+05 | 1,66E+05 | 1,86E+05 | 1,34E+05 | 9,37E+04 |
|  | 1,83E+05 | 1,18E+05 | 1,57E+05 | 1,52E+05 | 1,36E+05 | 8,65E+04 |
|  |  |  |  |  |  |  |
| PRDX3_DYGVLLEGSGLALR.2y7 | 2,69E+05 | 2,28E+05 | 2,01E+05 | 2,70E+05 | 3,34E+04 | 1,69E+05 |
|  | 2,63E+05 | 2,15E+05 | 1,88E+05 | 2,23E+05 | 2,63E+04 | 1,70E+05 |
|  | 2,36E+05 | 2,09E+05 | 1,31E+05 | 2,12E+05 | 2,54E+04 | 1,64E+05 |
|  |  |  |  |  |  |  |
| PRDX3_DYGVLLEGSGLALR.2y9 | 2,37E+05 | 1,64E+05 | 1,62E+05 | 2,23E+05 | 2,50E+04 | 1,35E+05 |
|  | 1,89E+05 | 1,72E+05 | 1,31E+05 | 1,99E+05 | 2,17E+04 | 1,38E+05 |
|  | 2,06E+05 | 1,81E+05 | 1,25E+05 | 1,64E+05 | 2,13E+04 | 1,34E+05 |
|  |  |  |  |  |  |  |
| PRDX3_DYGVLLEGSGLALR.2y10 | 3,05E+05 | 2,44E+05 | 2,33E+05 | 3,11E+05 | 3,70E+04 | 1,85E+05 |
|  | 2,78E+05 | 2,08E+05 | 2,05E+05 | 2,49E+05 | 3,30E+04 | 1,79E+05 |
|  | 2,75E+05 | 2,16E+05 | 1,72E+05 | 1,87E+05 | 2,74E+04 | 1,88E+05 |
